# Knowledge From London and Berlin: Finding Threads to a Functional HIV Cure

**DOI:** 10.3389/fimmu.2021.688747

**Published:** 2021-05-27

**Authors:** Jingyi Ding, Yanxi Liu, Yu Lai

**Affiliations:** ^1^ Hospital of Chengdu University of Traditional Chinese Medicine, Chengdu, China; ^2^ University of California, Los Angeles, Los Angeles, CA, United States; ^3^ School of Basic Medicine, Chengdu University of Traditional Chinese Medicine, Chengdu, China

**Keywords:** functional HIV cure, coreceptor-based hematopoietic stem cell gene therapy, combination antiretroviral therapy, milder conditioning regimens, shock and kill strategies

## Abstract

Despite the ability of combination antiretroviral therapy (cART) to increase the life expectancy of patients infected with human immunodeficiency virus (HIV), viral reservoirs persist during life-long treatment. Notably, two cases of functional cure for HIV have been reported and are known as the “Berlin Patient” and the “London Patient”. Both patients received allogeneic hematopoietic stem cell transplantation from donors with homozygous *CCR5* delta*32* mutation for an associated hematological malignancy. Therefore, there is growing interest in creating an HIV-resistant immune system through the use of gene-modified autologous hematopoietic stem cells with non-functional *CCR5*. Moreover, studies in *CXCR4*-targeted gene therapy for HIV have also shown great promise. Developing a cure for HIV infection remains a high priority. In this review, we discuss the increasing progress of coreceptor-based hematopoietic stem cell gene therapy, cART, milder conditioning regimens, and shock and kill strategies that have important implications for designing potential strategies aiming to achieve a functional cure for the majority of people with HIV.

## Introduction

At the end of 2019, an estimated 38.0 million people were suffering from human immunodeficiency virus (HIV) infection, and HIV remains a major issue in global public health, having claimed almost 33 million lives to date (1). Fortunately, widespread use of combination antiretroviral therapy (cART) has been effective in increasing the life expectancy of the patients infected with HIV with a significant reduction in morbidity and mortality, and thereby has successfully transformed HIV infection from a fatal disease into a manageable chronic health condition. Nevertheless, the latent HIV-1 reservoir is a major barrier to viral eradication, despite effective cART. Since protracted interruption of cART will lead to rebound of viral replication and recurrence of clinical symptoms, treatment must be life-long for most HIV-infected individuals.

The entry of HIV-1 into target cells requires the interaction of the viral surface envelope glycoprotein, gp120, with the CD4 glycoprotein and co-receptors including chemokine receptor CCR5 and CXCR4 on the surface of cell (2, 3). CCR5-using HIV-1 viruses (R5 viruses) are usually dominant in the early and chronic phases of HIV-1 infection. However, variants able to use CXCR4 (X4 viruses) or both CCR5 and CXCR4 (R5X4 viruses) can be isolated from approximately 40% to 50% of HIV positive individuals as the infection progresses to AIDS (4).

Although the expression of “functional cure” is contradictory and inconsistently employed across a wide array of research initiatives aimed at curing HIV, it has been used most frequently to refer to long-term ART-free HIV control, meaning restoring the immune system’s ability to combat infection (5). The “Berlin Patient” (6) and the “London Patient” (7) are two examples of patients that have been functionally cured of HIV. Both these cases utilized cell therapy with allogeneic hematopoietic stem cell transplantation (allo-HSCT) from donors with a homozygous *CCR5* delta32 mutation (*CCR5*Δ*32*) for an associated hematological malignancy. However, this road to a cure is formidable and unlikely to be a practical option for the vast majority of patients. Over recent decades, a *CCR5-* and/or *CXCR4*-based hematopoietic stem cell (HSC) gene therapy, as a more feasible approach, has undergone development. Additionally, the “shock and kill” strategy, of latency revival followed by drug or immune-mediated clearance of infected cells, has also been tested. These results lay a foundation for functional cure of HIV. Here, we review the increasing progress in this area and discuss the potential combination of coreceptor-based autologous HSC gene therapy and latent reservoir elimination strategies aiming to achieve HIV cure.

## Knowledge From Berlin and London

In 2009, a case report (the Berlin Patient) described a functional cure of HIV infection that followed treatment for acute myeloid leukemia (AML) myeloablative using allogeneic HSCT from a donor carrying homozygous *CCR5*Δ*32* (6). Ten years later, a second individual (the London Patient) was identified, who, following treatment for Hodgkin’s lymphoma, also experienced HIV-1 remission after allogeneic HSCT from a donor with homozygous *CCR5*Δ*32* (7). These two cases generated considerable inspiration for developing a cure for HIV. In contrast to other previous transplantation studies in HIV patients, both the patients underwent allo-HSCT from a human leukocyte antigen-matched homozygous *CCR5*Δ*32* donor for an associated hematological malignancy, whereas two “Boston Patients” (8) and one “Minnesota Patient” (9), experienced rebound viremia after receiving allo-HSCT in the absence of *CCR5*Δ*32*. These findings confirm that *CCR5*Δ*32* plays a critical role in viral suppression or eradication. Even though the latter three patients failed to achieve functional cure, temporary HIV-1 remission was still observed probably because of the graft-versus-host disease (GvHD) effect. A summary of differences between the Berlin and London Patients and other patients is provided in **Table 1**.

**Table 1:** Differences between the Berlin and London Patients and other Patients.

Patients	Malignancy types	ART regimen	Conditioning regimen	HSC donor	Viral load after HSCT	ART interruption	Viral remission	Viral rebound
Berlin Patient	Acute myeloid leukemia	EFV, FTC, TDF	HSCT #1: FLAMSA, CTX, ATG, TBI; HSCT #2: Ara-C, GO, TBI	10/10 HLA match; homozygous for *CCR5* delta*32*	Undetectable	Day of HSCT	Over 12 years	No
London Patient	Hodgkin lymphoma	EFV, FTC, TDF, RAL,RPV, 3TC, DTG	LACE, anti-CD52	9/10 HLA match; homozygous for *CCR5* delta*32*	Undetectable	16 months after HSCT	Over 3 years	No
Düsseldorf Patient	Acute myeloid leukemia	FTC, TDF, DRV, RAL, ABC, 3TC, DTG	Flu, Treo	10/10 HLA match; *CCR5* delta*32*	Undetectable	4 years after HSCT	NA	No
Minnesota Patient	Acute lymphoblastic leukemia	AZT/3TC IDV/rtv AZT/LAM, TDF/FTC, ATV/rtv, RAL, etravirine	RIC (Flu/Mel)	8/8 HLA-matched, ABO-matched; wild-type *CCR5*	Detectable at 56 days after HSCT. Undetectable at 91 days after HSCT	2 years after HSCT	288 days	Yes
Boston Patients	A: Hodgkin lymphoma	A: EFV, FTC, TDF, RAL, DRV/r	A: RIC chemotherapy (busulfan, Flu)	A: 7/8 HLA match; without *CCR5* delta*32*	A: Undetectable	A: 4.3 years after HSCT	A: 84 days	A: Yes
	B: Diffuse large B-cell lymphoma	B: EFV, FTC, TDF, NFV, ABC, RAL	B: RIC chemotherapy (busulfan, Flu)	B: 8/8 HLA match; without *CCR5* delta*32*	B: Undetectable	B: 2.6 years after HSCT	B: 225 days	B: Yes
Essen Patient	Anaplastic large-cell lymphoma	LPV/r, TDF, FTC, 3TC, ABC, RAL	ATG, CSA MTX	10/10 HLA match; homozygous for *CCR5* delta*32*	Undetectable	7 days before HSCT	20 days	Yes
Mississippi baby		AZT, 3TC, NVP, LPV/r; began receiving ART 30 hours after birth				18 and 23 months of age	27 months	Yes

EFV, efavirenz; FTC, emtricitabine; TDF, tenofovir disoproxil fumarate; RAL, raltegravir; RPV, rilpivirine; 3TC, lamivudine; DTG, dolutegravir; DRV/r, darunavir; ABC, abacavir; LPV/r, ritonavir-boosted lopinavir; NFV, nelfinavir; AZT, zidovudine; LAM, lamivudine; NVP, nevirapine; IDV, indinavir; rtv, ritonavir; ATV, atazanavir; Flu/Mel, fludarabine/melphalan; FLAMSA, fludarabine, Ara-C, and amsacrine; CTX, cyclophosphamide; ATG, anti-thymocyte globulin; TBI, total-body irradiation; Ara-C, cytarabine; GO, gemtuzumab ozogamicin; LACE, lomustine, Ara-C, cyclophosphamide, etoposide; Flu, fludarabine; Treo, treosulfan; RIC, reduced-intensity conditioning; CSA, cyclosporine-A; MTX, methotrexate; NA, not available.

The experience of the London Patient is quite similar to that of the Berlin Patient in many ways, but the differences between them have been noted (10). First of all, the Berlin Patient underwent two fully allogeneic HSCTs, including cyclophosphamide in conjunction with total body irradiation (TBI), whereas the London Patient received a less aggressive conditioning regimen, consisting exclusively of chemotherapy agents. In addition, the lymphocytes and host T cells of the Berlin Patient” were destroyed by antithymocyte globulin, whereas the London Patient received anti-CD52 for lymphodepletion. These reports provide a good starting point for further research to apply nongenotoxic conditioning regimens during allo-HSCT. The Berlin Patient interrupted antiretroviral treatment (ART) on the day of allo-HSCT, whereas in the case of the “London Patient”, ART was continued for 16 months after transplantation. With the longer period under the cover of ART and utilizing a less intensive conditioning regimen, the London Patient seems to have experienced a superior recovery and achieved the similar result of reservoir clearance.

Despite the remarkable curative potential of *CCR5*Δ*32* allo-HSCT, there are apparent limitations. These include the low incidence of the *CCR5*Δ*32* allele and the small chance of finding an appropriate donor, as well as toxicity related to conditioning regimens and risks associated with GvHD. Therefore, it is not always feasible for HIV patients without hematological malignancy to undergo this treatment, and transplantation using gene-edited autologous cells combined with milder conditioning regimens automatically become an alternative therapy.

## Combination Antiretroviral Therapy

In 2013, 14 HIV post-treatment controllers (PTCs) were noted with long-term virological remission for at least 24 months after the discontinuation of prolonged cART initiated within 10 weeks of a primary HIV infection (11). Unlike spontaneous HIV controllers, most PTCs did not exhibit the protective HLA B*27 or B*57 alleles but carried unfavorable risk-associated alleles HLA B*35 and HLA-B*07 (11, 12). A further analysis showed that both a low reservoir distribution in resting CD4+ T cells and the very small size of HIV reservoirs, induced by early and prolonged cART, were critical elements for the successful infection control off therapy (11). The very low HIV-1 DNA reservoir was unlikely to be replenished after the interruption of cART, leading to an extended control of viral replication (13).

With the London Patient, a combination of a less aggressive conditioning regimen and the graft-versus-reservoir (GvR) effect associated with allo-HSCT contributed to the reduction of viral reservoirs. It is reasonable to believe that a longer period covered by cART, as seen with the London Patient can effectively control virus replication, and thereby maintain a very low viral reservoir size until a functional HIV-resistant immune system is created *via* transplantation with HSCs homozygous for the *CCR5*Δ*32* mutation.

## *CCR5*-Targeted Gene Therapy for HIV

Gene therapy based on eliminating *CCR5* of HSC is a promising curative approach to reestablish the ravaged immune system and protect people from HIV invasion, perhaps ultimately eradicating the virus in patients with HIV. Currently, several gene editing techniques, including zinc finger nuclease (ZFN) (14), transcription-activator-like effector nuclease (TALEN) (15), and clustered regularly interspaced short palindromic repeats (CRISPR)-associated protein 9 (CRISPR/Cas9) technologies (16), have been developed and widely applied to induce genome modifications at specific target sites contributing to knockout of *CCR5* in CD4+ T Cells or HSCs.

### Zinc Finger Nuclease

In a preliminary study, ZFN-modified CD4+ T cells with disruption of *CCR5* were engrafted into NOD/SCID/IL2Rgamma null (NSG) mice (17). After 50 days of HIV-1 infection, more than 50% of CD4+ T cells in 8 out of 10 mice carried a disrupted *CCR5*; these mice also had lower plasma viremia and higher CD4+ T cell counts in their peripheral blood than those of mice engrafted with wild-type CD4+ T cells (17). Compared with CD4+ T cells, HSCs have an extended lifespan, and gene-modified autologous HSC therapy has become an attractive strategy for controlling or eliminating HIV infection. NSG mice were engrafted with ZFN-treated HSCs to produce *CCR5*-disrupted polyclonal multilineage progeny. Ten to twelve weeks after infection, HIV-1 RNA was undetectable in the gut lymphoid tissue of the mice (18). Additionally, a NSG mouse that was transplanted with *CCR5*-ZFN-modified adult hematopoietic stem/progenitor cells (HSPCs) and that was infected with HIV-1 had high levels of human CD4+ T cells and CD45+ cells (19). Moreover, a preclinical study used electroporation to develop a nonviral platform for the delivery of mRNA encoding *CCR5*-specific ZFN sequences; this resulted in 72.9% modification of HSPC-derived colonies, which contained biallelic disruption without the potential for tumorigenesis or leukemagenesis in NSG mouse model (20).

Early studies revealed that cytotoxicity of ZFNs is closely connected with target site overlap leading to apoptosis and cell death, which limited the completion of an associated clinical trial (21). Notably, substantial ZFN-induced off-target activity at *CCR2*, the closest relative of *CCR5* in the human genome, was revealed when editing the primary human cells (17). At present, retroviral vectors such as adenovirus vectors are widely used in gene approaches and can consistently deliver inducible ZFNs and reduce potential off-target risks (14). In spite of the high efficiency that lentiviral vectors provide for disruption of targeted genes in vector-treated cell lines and primary cells (22), foamy viral vectors are less likely to transactivate nearby genes and have less genotoxicity (23).

A number of completed or ongoing clinical trials have preliminarily demonstrated the feasibility and safety of ZFN-based *CCR5* disruption in CD4+ T cells or CD34+ HSPCs. A completed phase I clinical trial (ClinicalTrials.gov Identifier: NCT00842634) used autologous CD4+ T cells with *CCR5* disrupted by ZFN to significantly decrease the decline in circulating CD4+ T cells in comparison with that seen using unmodified cells (24); following a treatment interruption, the level of HIV DNA in the blood of most of the patients was observed to have decreased and HIV RNA became undetectable in a quarter of patients (24). These results demonstrate the efficiency of ZFN-based genetic modification and the safety of *CCR5*-modified autologous CD4+T cell infusions, although the small cohort sizes limit the conclusions reached. Another ongoing clinical trial (ClinicalTrials.gov Identifier: NCT02500849) released details that were discussed above (20). This pilot trial is expected to be completed in 2022, and the investigators hope to offer crucial data for clinical studies to develop a new strategy to complement the existing therapies. All completed or ongoing clinical trials using gene editing technologies are highlighted in **Table 2**.

**Table 2:** Clinical experiments of *CCR5*-based stem/progenitor cell or T cell therapy for HIV-1 infection.

Trial Number	Study Title	Tool	Date	Interventions	Status
NCT04201782	Long-Term Follow-up of HIV-Infected Subjects Treated With Autologous T-Cells Genetically Modified at the *CCR5* Gene by Zinc Finger Nucleases (SB-728-T or SB-728mR-T)	ZFN	2011–2031	Infusion of *CCR5*-disrupted SB-728-T or SB-728mR-T	Enrolling by invitation
NCT03617198	A Pilot Study of T Cells Genetically Modified by Zinc Finger Nucleases SB-728mR and CD4 Chimeric Antigen Receptor in HIV-infected Subjects	ZFN	2019–2025	Infusion of autologous T cells genetically modified to express a CD4 chimeric antigen receptor while also having ZFN-mediated disruption of the *CCR5* gene	Active, not recruiting
NCT02140944	Cord Blood Transplantation With *CCR5*Δ*32* Donor Cells in HIV-1 Infected Subjects Who Require Bone Marrow Transplantation for Any Indication and Its Observed Effects on HIV-1 Persistence		2015–2023	Transplantation with *CCR5*Δ*32* cord blood stem cells	Active, not recruiting
NCT02500849	A Pilot Study to Evaluate the Feasibility, Safety and Engraftment of Zinc Finger Nuclease (ZFN) *CCR5* Modified CD34+ Hematopoietic Stem/Progenitor Cells (SB-728mR-HSPC) in HIV-1 (R5) Infected Patients	ZFN	2015–2022	Infusion of *CCR5*-disrupted SB-728mR-HSPC after conditioning with busulfan	Active, not recruiting
NCT03164135	Safety and Feasibility Study of Allotransplantation of CRISPR/Cas9 *CCR5* Gene Modified CD34+ Hematopoietic Stem/Progenitor Cells in HIV-infected Subjects With Hematological Malignancies	CRISPR/Cas9	2017–2021	Transplantation of CD34+ hematopoietic stem/progenitor cells genetically modified at the CCR5 gene by CRISPR/Cas9	Recruiting
NCT02732457	Allogeneic Hematopoietic Stem Cell Transplantation in HIV-1 Infected Patients		2014–2024	Infusion of *CCR5*Δ*32* allogeneic HSCT in HIV-infected patients	Recruiting
NCT03666871	T-Cell Reinfusion After Interfering With Lymphocyte Binding Location of AIDS Virus Through Zinc-finger-nuclease Elimination of *CCR5* Receptors: The TRAILBLAZER Study	ZFN	2019–2024	Transplantation of autologous CD4+ T cells genetically modified at the *CCR5* gene by ZNF SB-728 versus	Recruiting
NCT00842634	A Phase I Study of Autologous T-Cells Genetically Modified at the *CCR5* Gene by Zinc Finger Nucleases SB-728 in HIV-Infected Patients	ZFN	2009–2013	Infusion of *CCR5*-disrupted CD4+ T Cells	Completed
NCT01252641	A Phase 1/2, Open Label, Single Infusion Study of Autologous T-Cells Genetically Modified at the *CCR5* Gene by Zinc Finger Nucleases (SB-728-T) in HIV-Infected Subjects	ZFN	2010–2015	Infusion of *CCR5*-disrupted SB-728-T	Completed
NCT01044654	A Phase 1 Dose Escalation, Single Dose Study of Autologous T-Cells Genetically Modified at the *CCR5* Gene by Zinc Finger Nucleases SB-278 in HIV-Infected Patients Who Have Exhibited Suboptimal CD4+ T-Cell Gains During Long-Term Antiretroviral Therapy	ZFN	2009–2014	Infusion of *CCR5*-disrupted SB-728-T	Completed
NCT01543152	A Phase I, Open-Label Study to Assess the Effect of Escalating Doses of Cyclophosphamide on the Engraftment of SB-728-T in Aviremic HIV-Infected Subjects on HAART	ZFN	2011–2017	Infusion of *CCR5*-disrupted SB-728-T after conditioning with cyclophosphamide	Completed
NCT02225665	A Phase 1/2, Open-Label Study to Assess the Safety and Tolerability of Repeat Doses of Autologous T-Cells Genetically Modified at the *CCR5* Gene by zinc finger Nucleases in HIV-Infected Subjects Following Cyclophosphamide Conditioning	ZFN	2014–2018	Infusion of *CCR5*-disrupted SB-728mR-T after conditioning with cyclophosphamide	Completed
NCT02388594	A Phase I Study of T-Cells Genetically Modified the *CCR5* Gene by Zinc Finger Nucleases SB-728mR in HIV-Infected Patients, with or without the *CCR5* Delta-32 Mutation, Pretreated With Cyclophosphamide	ZFN	2015–2019	Infusion of autologous CD4+ T cells genetically modified at the *CCR5* gene by ZFN SB-728mR with or without cyclophosphamide	Completed

(The data is available at https://clinicaltrials.gov/ct2/home) HSCT, hematopoietic stem cell transplantations; ZFN, zinc finger nuclease. CRISPR/Cas9, clustered regularly interspaced short palindromic repeats-associated protein 9.

### Peptide Nucleic Acids

Peptide nucleic acids (PNAs) have emerged as a powerful biomolecular tool to use for mutation correction at targeted locations in cell culture and *in vivo*. These form a triplex structure with DNA capable of producing specific genome modification and inducing DNA repair (25). In 2011, *CCR5*-targeted PNAs were transfected into human cells, and a termination codon was introduced using a 60-nucleotide antisense donor DNA, mimicking the naturally occurring *CCR5*Δ*32* mutation (26). This study resulted in 2.46% *CCR5* modification, and the off-target rate in *CCR2* was less than 0.057%. In contrast, the off-target rate of ZFN was 5.39% (17), almost two orders of magnitude more than that of the PNAs. This means that PNAs are more effective for increasing editing potency and reducing off-target toxicity.

### Transcription-Activator-Like Effector Nucleases

TALENs act similarly to ZFNs but utilize non-specific FokI as a nuclease effector fused to a DNA-binding domain (27). The efficiency of a *CCR5*-specific TALEN in genome editing activity comparable with that of ZFN, causing minimal off-target activity at the *CCR2* locus and without affecting the cell cycle (28). TALENs have significant less cytotoxicity than and may be a better alternative to ZFNs.

Cell culture using the TALEN showed that the technique achieved cleavage efficiency more than 50% to reproduce homozygous *CCR5*Δ*32* mutations in CD4+-U87 cells with no selective pressure; the *CCR5* to *CCR5*Δ*32* recombination occurred in 8.8% in targeted cells, which were then resistant to HIV infection (29). This study put forward a broad prospect of using TALENS to generate site-specific, size-controlled, and homozygous DNA deletions within mammalian genomes. Additionally, after transferring TALEN-encoding mRNA into CD4+ T cells, the *CCR5* alleles were disrupted by 90%, whereas the off-target activity was absent and the edited cells proliferated normally (30). As a result, TALEN-mediated *CCR5* disruption of CD4+ T cells is paving the way for clinical applications in CD34+ HSCs.

### Clustered Regularly Interspaced Short Palindromic Repeats-Associated Protein 9

The CRISPR/Cas9 system is a recently developed tool for genome editing that utilizes a predesigned guide RNA (gRNA) sequence to identify a precise target of the genome (31). In a comparison with TALEN, the CRISPR/Cas9 system produced 4.8-fold more gene editing when targeting the same region of the *CCR5* gene (32). With a greater efficiency, higher accuracy, and lower costs in editing, the CRISPR/Cas9 system is more effective than either ZFN- or TALEN-based editing and is generating considerable excitement.

CRISPR/Cas9 was applied by Fernando et al. using *CCR5*Δ*32* genetically edited induced pluripotent stem cells combined with PiggyBac technology to obtain CD34+ HSCs that can offer resistance to the CCR5-tropic viruses and to some extent to CCR5/CXCR4 dual-tropic viruses (33). After transplanting the CRISPR/Cas9-modified HSPCs into NSG mice, the *CCR5* deletions were validated by polymerase chain reaction analysis and the combination of CRISPR/Cas9 with a dual gRNA effectively ablated *CCR5* in CD34+ HSPCs such that the biallelic inactivation frequencies reached 42%; additionally, the off-target mutation events accounted for approximately 0.6% of all captured off-target sites (34). Faced with HIV-1 challenge, these human CD4+ T cells remained stable and the virus RNA level decreased compared with that in the control mice, indicating that the CRISPR/Cas9-modified HSPCs produced *CCR5*-ablated CD4+ cells that consistently contributed to HIV-1 resistance *in vivo* (35).

According to a study on Mauritian cynomolgus macaque embryos, *CCR5* disruption was detected in more than half of the embryos, and 36.7% of the embryos contained biallelic deletions introduced by dual gRNAs targeting *CCR5* (36). Research in simian immunodeficiency virus-infected rhesus monkeys has used the CRISPR/Cas9-*CCR5* lentivirus to modify the autologous peripheral HSPCs of three macaques. After transplantation, the *CCR5*-edited CD4+ T cells consistently increased in abundance and achieved basic reconstruction of the hematopoietic system. Furthermore, plasma-borne virus in one of the three monkeys became undetectable after ART cessation for seven months, and the off-target events remained undetectable (37). However, despite its safety and efficacy, another two rhesus monkeys displayed a viral rebound after ceasing ART. Further research is needed to delimitate a better non-myeloablative dosage as well as the optimal time to cease the ART.

One of the most commonly used Cas9 proteins, adapted from *Streptococcus pyogenes* (SpCas9), has high specificity but is limited by its size. Fortunately, the small-sized *Staphylococcus aureus* (SaCas9) has an advantage of high delivery efficiency compared with that of SpCas9 (38, 39). Recent evidence also suggests that *CCR5*-editing in HSCs using lentiviral vector-mediated CRISPR/SaCas9 maintains potential multilineage ability (39), indicating that SaCas9 can mediate genome editing with high specificity and low toxicity.

Recently, a clinical study (ClinicalTrials.gov Identifier: NCT03164135) was performed to evaluate the feasibility and safety of CRISPR/Cas9 *CCR5*-modified HSPC transplantation. HIV-infected individuals with acute lymphoblastic leukemia (ALL) were treated by *CCR5*-ablated HSPCs using CRISPR/Cas9 after conditioning with cyclophosphamide and TBI. During the treatment, hematological malignances were in complete remission. Nevertheless, the gene-editing efficiency was evaluated as 17.8% before transplantation and the frequencies of *CCR5*-edited cells ranged between 5.20% and 8.28% after transplantation. Furthermore, the *CCR5*-edited frequencies of CD4+ cells (approximately 2%) and CD8+ cells (approximately 1%) in peripheral blood were far lower than that of other cells, which was not sufficient to achieve the aim of an HIV cure (40). The underlying mechanisms for the poor engraftment of *CCR5*-modified CD4+ and CD8+ cells utilizing CRISPR/Cas9 remains unclear, and should be elucidated further. Subsequent high-quality research is required to improve the efficiency of CRISPR/Cas9-mediated *CCR5* ablation in human HSCs.

## *CXCR4*-Targeted Gene Therapy for HIV

Homozygosity for *CCR5*Δ*32* does not confer absolute HIV-1 resistance. Several reports have indicated that persons with the *CCR5*Δ*32*/Δ*32* genotype are also infected by X4 or R5X4 viruses (41–44). One intriguing notion is aimed at genome editing or suppression of *CXCR4*, as the same gene therapies that have been applied to editing *CCR5* can also be used for *CXCR4*. In a humanized mouse model, Wilen et al. used a specific ZNF to disrupt *CXCR4* in human CD4+ T cells, which were subsequently protected from attack by X4-tropic HIV-1 (45). In contrast with stable lentiviral expression of short-hairpin RNAs targeting *CXCR4* mRNAs, a transient adenovirus delivered ZFN to disrupt genomic *CXCR4*; this provided more durable resistance to viruses when CD4+ T cells were challenged with HIV-1 that utilizes CXCR4 for entry (46). The CRISPR/SaCas9 system has also been developed as a more efficient genome editing tool to target *CXCR4* of primary CD4+ T cells because of the smaller size in comparison with CRISPR/SpCas9. Using selected single-guided RNA and lentivirus expressing SaCas9, *CXCR4*-modified primary CD4+ T cells were generated that proliferated normally and successfully established resistance to HIV-1 infection (47).

A study has also disrupted both *CCR5* and *CXCR4* in human CD4+ T cells using ZFNs; these co-receptor negative cells proliferated normally and were protected from *CCR5*- and *CXCR4*-utilizing strains of HIV-1 both *in vitro* and *in vivo* (48). Meanwhile, CRISPR/Cas9-ablated *CCR5* and *CXCR4* genes also showed outstanding protection from both *CCR5*- and *CXCR4*-utilizing strains in CD4+ cell lines, with efficacies up to 55% for *CCR5* and 36% for *CXCR4* (49). Studies showed that there were negligible off-target effects, absent genotoxicity, and normal cell propagation (50, 51).

Nonetheless, *CXCR4* is of significant importance in multiple tissues and cell types such as the hematopoietic, immune, and nervous systems (52). Moreover, *CXCR4* is required to maintain the physical function of HSCs, and, unlike the effects of *CCR5* disruption in HSCs, deficiency of *CXCR4* causes movement and migratory disorders of hematopoietic cells (53). Therefore, the direct gene-disrupted therapy of HSCs may not be suitable for *CXCR4*. Interestingly, combined CRISPR/Cas9 with piggyBac transposon technology constructed a *P191A* mutant, one of the natural mutants of *CXCR4*, without any redundant DNA or off-target effects as an alternative to *CXCR4* knockout (54). Replacing *CXCR4* mutation with that in *P191A* avoided undesirable side effects of CXCR4 deficiency but abrogated HIV-1 replication by up to 59.2% (55).

In addition, there is accumulating evidence that antagonists of CXCR4 can feasibly prevent cells from HIV-1 attack. According to a new study, the pepRF1 peptide, derived from dengue virus capsid protein, can specifically act against the CXCR4-tropic strains and is more effective in inhibiting HIV-1 than other approved peptide-based inhibitors (56). Utilizing computational and thermodynamic parameters, a number of Himalayan bioactive molecules were also identified as potential inhibitors of the CCR5 or CXCR4 co-receptors (57). Specifically, dactylorhin-A and butyl 2-ethylhexyl phthalate are closely related to CCR5 while amarogentin, dactylorhin-E, and pseudohypericin exhibit a higher binding affinity with CXCR4 (57).

## Milder Conditioning Regimens

In autograft transplantation of HIV therapy, conditioning treatment is used to create space for new gene-edited cells and eliminate a portion of viral reservoirs. However, the goal here is not to ablate and reestablish the entire hematopoietic system (58). For the non-malignant HIV/AIDS patient, low intensity conditioning procedure is required to reduce the risk of mortality.

Considerable efforts are being exerted to generate new milder or non-myeloablative conditioning regimens. Monoclonal antibody (mAb) therapy is a form of immunotherapy that can target the hematologic system. Combined radiation therapy with mAb, the commonly known radioimmunotherapy (RIT), is an efficient strategy with a high degree of accuracy. In a murine experiment, anti-CD45 RIT successfully replaced TBI (59); additionally, a CD45-targeted antibody-radionuclide conjugate is being applied to treat hematologic malignancies in an ongoing trial (60). Furthermore, 213Bi-anti-CD45 has been used to target hematopoietic cells in hematologic neoplasms, whereas 213Bi-anti-TCRab was applied to target T cells in non-malignant diseases (61).

In an animal study, 11 dogs that received RIT with 213Bi-anti-TCRab experienced some degree of pancytopenia, although to a lower intensity than that found dogs treated with 213Bi-anti-CD45. After receiving marrow grafts from dog leukocyte antigen-identical litter-mates, all the dogs had rapid hematopoietic recoveries, and 10 dogs showed continuing mixed chimerism until the end of the study (61). These results demonstrate the feasibility of replacing TBI with RIT for non-myeloablative HSCT.

Recently, Canakci et al. used an anti-CD4 conjugated polymer nanoparticle that selectively targeted a cytotoxic drug molecule to the primary CD4+ T cells or CD4 lymphoma cells in a T cell ALL mouse model. With higher accuracy, the conjugation of anti-CD4 to a nanogel is far beyond conventional therapy but needs much less antibody to reach an equal outcome (62). Furthermore, the CD45-saporin (SAP) can be used without irradiation, avoids anemia and neutropenia and spares thymic niches and the bone marrow (63), and therefore is a promising alternative non-genotoxic conditioning method for curing HIV-1. A recent study reported that anti-CD117 specifically targeted and depleted HSCs *in vivo* with minimal toxicity and high efficiency and that a single dose of CD117-SAP depleted more than 99% of HSCs (64). In the future, mAbs that specifically target HSC surface receptors probably play an important role in the elimination of HSCs, and increase the engraftment of their gene-modified counterparts. These results cast light on finding new extraordinary non-myeloablative conditioning treatments for patients with non-malignant HIV/AIDs who are undergoing gene-modified autologous HSCT, and future research should continue to explore HSC-targeted mAbs and combine the antibody-based cell killing with irradiation or nanotechnology to generate more selective conditioning regimes.

## Shock and Kill Strategy

Due to the GvHD-related GvR effect, latent reservoir elimination and a temporary HIV-1 remission were observed for the Boston Patients (8) and the Minnesota Patient (9). However, GvHD is absent when transplanting autologous cells into HIV patients. The commonly known “shock and kill” approach aimed at the latent HIV-1 reservoir is an important complement for autologous HSCT. This strategy involves reactivating and eradicating virus that can hide in CD4+ T cells when the retroviral gene is integrated into their DNA. The first step of this process requires using latency reversal agents (LRAs) (summarized in **Table 3**) that induce expression of latent HIV virus.

**Table 3:** Summary of Latency-Reversal Agents.

Latency-Reversal Agents	Class	Stage	References
Romidepsin (FK228), Vorinostat (SAHA), Panobinostat(LBH589)	Histone deacetylase inhibitors	Clinical trial	NTC02092116 (65), NCT02092116 (65, 66), NCT01933594 (67, 68), NCT01319383 (68–71), NTC02092116 (65), NCT01365065 (72, 73), NCT03803605 (74), NCT02336074 (75), NCT01680094 (69, 76), NCT02471430 (76)
Belinostat (PXD101), Givinostat (ITF2357), Nanatinostat(CHR-3996)	Histone deacetylase inhibitors	*In vitro*	(77, 78)
Nicotinamide	Histone deacetylase inhibitors	*Ex vivo*	(79)
JQ1, I-BET151, I-BET, MS417, UMB-136, OTX015, CPI-203, RVX-208, PFI-1, BI-2536, BI-6727, Apabetalone	Small-molecule bromodomain inhibitors	*In vitro*	(80–88)
GSK-343, EPZ-6438 (Tazemetostat)	Small-molecule histone methyltransferase inhibitors targeted to EZH2	*In vitro*	(89)
UNC-0638	Small-molecule histone methyltransferase inhibitors targeted to EHMT2	*In vitro*	(89)
EED226, A-395	Small-molecule histone methyltransferase inhibitors targeted to EED	*In vitro*	(90)
DZNep	Lysine-specific histone methyltransferase inhibitor targeted to EZH2	*In vitro*	(91, 92)
BIX-01294	Lysine-specific histone methyltransferase inhibitor of the HKMT G9a	*In vitro*	(79, 92)
Chaetocin	Lysine-specific histone methyltransferase inhibitor of the HKMT SUV39H1	*In vitro*	(79, 92)
5-AzaC, 5-AzadC	DNA methylation inhibitors	*In vitro*	(93)
Ingenol, Bryostatin-1	Protein kinase C activators	Clinical trial	NCT04503928 (94, 95), NCT02531295 (96–97), NCT02269605 (98)
Prostratin	Protein kinase C activators	*In vitro*	(99)
Tat-R5M4	Toll-like receptor agonist	*In vitro*	(100)
Pam3CSK4	Toll-like receptor 1/2 agonist	*In vitro*	(101)
GS-9620	Toll-like receptor 7 agonist	Clinical trial	NCT02858401 (102)
GS-986	Toll-like receptor 7 agonist	*In vivo*	(103)
R-848	Toll-like receptor 7/8 agonist	*In vitro*	(104)
3M-002	Toll-like receptor 8 agonist	*In vitro*	(104)
MGN 1703	Toll-like receptor 9 agonist	Clinical trial	NCT02443935 (105, 106)
CPG 7909	Toll-like receptor 9 agonist	Clinical trial	NCT00562939 (107)
nivolumab	Monoclonal antibodies targeting PD-1	Case report	(108)
pembrolizumab	Monoclonal antibodies targeting PD-1	*In vitro*	(108)
Ipilimumab	Monoclonal antibodies targeting CTLA-4 (CD152)	Case report	(109)
BMS-936559	Monoclonal antibodies targeting PD-L1	Clinical trial	NCT02028403 (110)
galectin-9	Human carbohydrate-binding immunomodulatory protein	*In vitro*	(111)
Procyanidin trimer C1	MAPK agonist	*In vitro*	(80)
Maraviroc	CCR5 antagonist	Clinical trial	NCT01365065 (112), NCT00795444 (113)
N-803	Interleukin-15 Superagonist	*In vivo*	(114)
AZD5582, Ciapavir (SBI-0953294)	Activator of the non-canonical NF- κB pathway	*In vivo*	(115, 116)
Disulfiram (C10H20N2S4)	Activators of Akt signaling pathway	Clinical trial	NCT01944371 (117)

NF- κB, Nuclear Factor- κB; HKMT, histone-lysine methyltransferase; EHMT2, euchromatic histone-lysine N-methyltransferase 2; PD-1, programmed cell death protein; CTLA-4, cytotoxic T lymphocyte antigen 4; PD-L1, programmed cell death protein ligand 1.

### Histone Deacetylase Inhibitors

Histone deacetylase inhibitors (HDACis) have emerged as a primary approach for boosting the provirus out of latency, without impacting CD4+ T cell activation (118). HDACis prevent the deacetylation of histone marks and elicit changes in chromatin structure, resulting in chromatin decondensation and gene transcription. Current HDACis include valproic acid, romidepsin, vorinostat, panobinostat, belinostat, and givinostat, several of which are actively being tested in clinical trials (119). A completed phase II clinical trial (ClinicalTrials.gov Identifier: NCT01365065) showed that vorinostat was well tolerated. After receiving vorinostat orally, plasma cell-associated unspliced (CA-US) HIV RNA in up to 90% of the participants was significantly and sustainably induced (73). In addition, extended follow-up for two years demonstrated that there were no adverse safety effects or virological changes in participants on ART following vorinostat treatment (72). In a phase I/II clinical trial (ClinicalTrials.gov Identifier: NCT01680094), panobinostat has been investigated as a promising candidate drug for HIV latency activation *in vivo*, although this needs to be combined with other LRAs to improve the efficiency (76). A more recent placebo-controlled phase I/II randomized trial (ClinicalTrials.gov Identifier: NCT01933594) reported that although romidepsin infusion was safe and displayed pharmacodynamic effects on CD4 + T cells, this did not effectively activate HIV-1 expression, as the plasma viremia or cell-associated unspliced HIV-1 RNA was not increased compared with that in the placebo (67).

The modest and transient activation of virus from HIV latency by HDACis is, however, unable to thoroughly clear the reservoir. None of the current HDACis tested have effectively reduced the number of latently infected cells or decreased the latent-reservoir size in ART-treated patients. The toxicity of HDACis targeting to immune cells is also becoming an obstacle to eradicating the HIV reservoir, which has caused wide public concern. Although two class I-selective HDACis, romidepsin and nanatinostat, significantly induced viral transcription *ex vivo*, the production of viral particles or antigens from primary CD4^+^ T cells was not facilitated. Both these HDACis exhibited toxicity to HIV-specific CD8+ T cells *in vitro* (78). Meanwhile, romidepsin and panobinostat were also shown to be highly cytotoxic for both CD8+ T cells and CD4+ T cells (120), which play important roles in HIV latency eradication. In addition, viral sequence variation also affects the ability of HDACis to reactivate latency (121).

### Bromodomain and Extra-terminal Domain Inhibitors

Bromodomain and extra-terminal domain inhibitors (BETis) are an identified class of LRAs that are promising anti-cancer drugs and that have been developed to purge latent HIV-1. BET inhibitors, including JQ1, MS417, I-Bet, and I-Bet151, have reactivated HIV from latency *via* the action of the positive transcription elongation factor b, a subject of acetylation, in a model of HIV latency (82, 122). Furthermore, I-Bet151 was investigated in humanized mice treated with suppressive cART (81). In this case, I-Bet151 did not have any observed significant toxic effect on the treated mice, and nine days after I-Bet151 initiation, HIV-1 viremia rebounded and was associated with a higher HIV-1 RNA level in different organs. This study also indicated that BETi reversed HIV-1 latency preferentially in monocytic cells rather than T cells.

### Histone Methyltransferase Inhibitors

Polycomb repressive complex 2 (PRC2) plays a crucial role in HIV transcriptional regulation and consists of three core components, EZH2, EED, and SUZ12 (123). The catalytic subunit EZH2, an extensively studied histone methyltransferase, methylates histone H3 lysine 27 (H3K27) and plays a pivotal role in HIV-1 latency. Histone methyltransferase inhibitors (HMTis), such as GSK-343 and EPZ-6438, targeted to EZH2 represent new tools for reducing the pool of latent reservoirs (89). Substantial proviral reactivation was observed using low doses of GSK-343 and EPZ-6438 alone in three polarized cell compartments (Th17, Th1, and Th2 cells) infected with vesicular stomatitis virus G-pseudotyped HIV-1. EED can enhance the activity of EZH2 (123). The activity of PRC2 was inhibited in both cell line and primary CD4+ T cell models of latency, following the binding of the allosteric histone H3K27me3 methyltransferase inhibitor EED226 to EED, demonstrating latency reversal activity (90). Meanwhile, euchromatic histone-lysine *N*-methyltransferase 2 (EHMT2) was necessary for establishing and maintaining HIV-1 proviral silence in primary cells in addition to the activity of PRC2 (89). The EHMT2 inhibitor UNC-0638 has been identified as a strong latency-reversing candidate in resting memory T cells isolated from cART-treated HIV-1 patients and in a primary Th17-cell model of HIV latency (89). At present, there are no BETis- or HMTis-based trials for HIV infection approved by US Food and Drug Administration. Future studies are therefore warranted to determine the efficacy of BETis and HMTis in HIV-infected patients under ART and identify the underlying mechanisms.

### Protein Kinase C Agonists

Due to the incomplete success of epigenetic LRAs in mediating pervasive and robust latency reversal, more effective strategies are required to reverse latency through the use of cellular signaling pathways. The protein kinase C agonists (PKCas) showed promising efficiency in reactivating HIV latency, *via* activating IκB kinase and degrading IκB to enhance NF-κB signaling (124). Small-molecule PKCas, e.g., ingenol (125), bryostatin-1 (126), and prostratin (127) have been studied *in vitro* or *ex vivo* to induce HIV expression and T cell activation. Prostratin was shown be superior in *in vitro* testing to bryostatin-1 in enhancing natural killer (NK) cells to clear the HIV-1 reservoir (128). In cell culture, both prostratin and bryostatin-1 were less cytotoxic to CD4+ T cells and CD8+ T cells compared with the cytotoxicity of panobinostat and romidepsin (120). The potency and safety of bryostatin-1 has been assessed in a double-blind phase I clinical trial (98). Although bryostatin-1 was well tolerated in all twelve participants, it did not significantly activate PKC or reactivate HIV in latently infected cells. However, the dose-related side effects of PKCas have raised concerns (129, 130), limiting preclinical and clinical studies. Higher doses cannot be feasibly used to increase the effect of PKCas as the active concentrations may be too toxic. Further study investigating the underlying mechanism is therefore warranted.

Recent studies have focused on the combination of PKCas and other different LRA families to enhance the activity of HIV-1 latency-reversing compared with the effects of single agents. Ingenol plus the HDACi romidepsin was certified as the most potent combination of LRAs in J-Lat cells. This induced a reactivation level up to 35% of GFP+ cells and increased p24 production and HIV transcription in most CD4+ T cell subsets (131). Notably, a combination of the BETis CPI-203 with prostratin prevented potentially severe clinical side effects related to HIV-1-induced “cytokine storm” and exhibited synergism in latent HIV-1 reactivation (85). Furthermore, a plant-derived tricyclic β-carboline alkaloid, harmine, enhanced the activity of ingenol A, leading to boosting of HIV reactivation (132).

### Toll-Like Receptor Agonists

One of the explanations for the failure of several LRAs in HIV latency clearance is that these LRAs cannot contribute to immune response or viral-mediated killing of reactivated cells. Recently, there has been growing interest in using toll-like receptor (TLR) agonists that possess potential dual effects as LRAs and immunomodulators to cure HIV-1. In humans, the TLR family comprises 10 members that recognize different pathogen-associated molecular patterns (133). TLRs are expressed on the surface of several cell types, including CD4+ T cells, which harbor the majority of HIV latent reservoirs. TLR agonists are capable of increasing immune activation, augmenting pre-existing adaptive ones, and playing crucial roles in inducing innate antiviral responses. Several TLR agonists, such as those to TLR2, 7, 8, and 9, have been investigated as LRAs. In the latently infected monocytic cell lines OM10 and U1, a TLR7/8 agonist R-848 activated HIV production (134), *via* triggering of TLR8 alone (104). Through a NF-κB and NFAT-dependent pathway, Pam3CSK4, a TLR1/2 agonist, was able to significantly reactivate HIV-1 transcription in a cell-cultured model of latency using central memory cells and also in resting CD4+ T cells isolated from aviremic HIV-1 infected donors without T cell activation or proliferation (101). The TLR9 agonist MGN1703 (135) and the TLR7 agonist GS-9620 (vesatolimod) (136, 137) increased HIV RNA levels and induced potent antiviral responses specific to HIV in peripheral blood mononuclear cells isolated from aviremic donors on suppressive ART.

A double-blind randomized controlled vaccine trial (ClinicalTrials.gov Identifier: NCT00562939) investigated the effects of TLR9 agonist CPG 7909 on the HIV latent reservoir and HIV-specific immunity. This study revealed that CPG 7909 strengthened the immune-mediated clearance of HIV-infected cells and decreased the proviral load compared with that in the control group (107). In another open-label, single-arm clinical trial (ClinicalTrials.gov Identifier: NCT02443935), 13 males and two females under suppressive ART received 60 mg MGN1703 subcutaneously twice a week for four weeks. Treatment with MGN1703 increased the activation of plasmacytoid dendritic cells, enhanced activation of cytotoxic NK cells and CD8+ T cells, upregulated cytokine levels, and induced plasma HIV RNA (106). In a multicenter, placebo-controlled, double-blind trial (ClinicalTrials.gov Identifier: NCT02858401), vesatolimod was shown to be well tolerated with no adverse events at doses ranging from 1 to 12 mg; immune stimulation was observed at doses above 4 mg, providing a rationale for future trials in people with HIV (102).

### Immune Checkpoint Inhibitors

Latent HIV infection is enriched in memory CD4+ T cells that express multiple immune checkpoint (IC) molecules, including programmed cell death protein 1 (PD-1), programmed cell death protein ligand 1 (PD-L1), cytotoxic T-lymphocyte-associated protein 4 (CTLA-4), T cell immunoreceptor with Ig and ITIM domains, and T cell immunoglobulin domain, and mucin domain-3 (138–140). Immune checkpoint inhibitors are described as a form of mAb that block the IC and act on silent HIV-1 in latently infected cells (140). Administration of nivolumab, directed to PD-1 in an HIV-infected and ART-treated individual with metastatic melanoma immediately increased the level of CA-US HIV RNA (108). One ART-treated patient infected with HIV received ipilimumab, which inhibits binding of CTLA-4 to its ligands CD80 and CD86, for treatment of disseminated melanoma. This profoundly increased the level of CA-US HIV RNA, whereas the level of plasma HIV RNA declined subsequently with an increase predominantly in total memory and effector memory CD4+ T cells (109). In a phase I study of BMS-936559 that targeted PD-L1 (ClinicalTrials.gov Identifier: NCT02028403), HIV-1-specific CD8+ responses, predicted by *ex vivo* response from a subgroup of participants receiving single low-dose BMS-936559 infusions, appeared to be enhanced compared with those in participants who received placebo (110). Additionally, in a new clinical trial (ClinicalTrials.gov Identifier: NCT02408861), participants with HIV associated classical Hodgkin lymphoma or solid tumors received ipilimumab and nivolumab (anti-PD-1). An increase in CA-US HIV RNA following infusion was noted, whereas anti-PD-1 alone had no effect on HIV-latency or the latent HIV-reservoir (141).

### Interleukin

Several cytokines, interleukin (IL)-2 (142), IL-7 (143), and IL-15 for instance, share the same γ chain, and are being used in the field of HIV treatment to act not only as an alternative immunomodulatory treatments to cART but also as therapeutic applications to play a putative role in latent reservoir clearance. A latency-reactivating strategy used the human IL-15 superagonist N-803 (ALT-803) in combination with MT807R1 (anti-CD8α) or CD8b255R1 (anti-CD8β) to selectively deplete CD8+ lymphocytes. This resulted in a robust and persistent HIV latency-reversing activity in simian immunodeficiency virus (SIV)-infected macaques on ART, and the results in non-human primates were concordant with those in humanized mice (114, 144). However, N-803 is not sufficient by itself to reverse virus latency and clear the reservoir *in vivo*. Subcutaneous dosing of N-803 to non-human primates activated and mobilized both SIV-specific CD8+ T cells and NK cells from peripheral blood to lymph nodes, but only minimal activation of memory CD4+ T cells was observed during the N-803 administration period. Meanwhile, there were no appreciable differences between N-803-treated and untreated rhesus macaques in the viral RNA content in the lymph node resident CD4+ T cells, number or magnitude of plasma viremia timepoints, or size of the viral DNA cell-associated reservoir (145).

### Other Agents

Importantly, unique broadly neutralizing antibodies (bnAbs) can naturally evolve in some HIV-infected people, with the ability to hinder HIV-1 infection by selectively targeting vulnerable sites on the viral envelope protein that mediates virus entry into susceptible CD4+ T cells (146). Recently, cocktails of bnAbs or multispecific antibody that simultaneously encodes different bnAb domains have been intensively studied for both therapy and prevention of HIV (147–149). These findings also support a potential role of bnAbs in the shock and kill strategy. To examine this approach, an anti-HIV bnAb, known as PGT121, combined with a toll-like receptor 7 agonist (GS-9620) as a latency reversing agent was administrated in simian/human immunodeficiency virus-infected monkeys (150). After ART interruption, rebound was observed in fewer animals receiving both the latency reversing agent and the experimental antibody than in those receiving either alone or neither, implying that bnAb treatment together with innate immune stimulation during ART suppression might effectively shrink the size of the viral reservoir. Meanwhile, definitive proof came from a phase II, single-center, open-label study indicated that maraviroc, a CCR5 antagonist, can induce latent HIV-1-transcription in resting CD4+ T cells from HIV-1-infected patients on suppressive ART through the activation of the transcription factor nuclear factor-κB by downstream signaling after binding to CCR5 (112). Then, maraviroc could be utilized, in addition to as an antiretroviral agent for HIV infection, as a part of LRAs. A follow-up initial clinical trial that recruited three patients explored additional virologic parameters in stored samples and evaluated the time to viral rebound during analytical treatment interruption (ATI) (151). Maraviroc spurred an increase in cell-associated HIV RNA during the administration of the drug, and the HIV-specific T cell responses, mainly from CD8+ T cells, were slightly enhanced in two out three patients after maraviroc intensification and ATI intervention. Nevertheless, there was a disappointingly rapid rebound of viremia after ART discontinuation. It is likely that maraviroc as an LRA is not potent enough and combinations of drugs that can efficiently remove free virus particles should be considered.

Unfortunately, a recent study has shown that the majority of the intact HIV-1 viral reservoir is not inducible in ART-suppressed HIV-infected individuals (152). Among all the LRAs tested, none has wiped out HIV-1 latency or affected viral rebound persistently after the interruption of ART.

## Conclusions

Despite the universal use of cART, HIV still continues to globally pose a major public health challenge. A cure for HIV is the ultimate goal that allows HIV-infected people to be free of disease without requiring antiretroviral therapy. The past decades have witnessed considerable advances from the understanding the pathogenesis of HIV to applying various therapeutic strategies for HIV disease. Knowledge from the London and Berlin Patients has important implications not only for identifying the potential factors implicated in long-term HIV remission, which orchestrate the elicitation of a long-standing antiviral effect, but also for providing direct evidence that current monotherapy is not sufficient to achieve an HIV cure. It is worth noting that in 2021 two gene therapy trials for sickle cell disease (ClinicalTrials.gov Identifier: NCT03282656) have been halted since two patients have developed AML and myelodysplastic syndrome, respectively (153), which provides insights into the safety of gene-modified autologous HSC therapy for HIV. Moreover, the shock and kill strategy is a promising, but not the sole, method involved in potential combined treatment strategies towards a functional cure of HIV. Interventions such as “block and lock”, aiming at the transcriptional and epigenetic silencing of proviruses, and “reduce and control”, diminishing the size of viral reservoir and controlling replication of this residual virus, have also flourished with the intent to induce a state of sustained ART-free HIV control. More importantly, recent data shows that CD4+ T cells expressing an HIV-specific chimeric antigen receptor can directly control HIV replication and augment the virus-specific CD8+ T cell response in humanized mice (154). Future studies are needed to continue to unravel the underlying molecular mechanism of these two cases and carefully determine the related parameters in situations that involve cART, HSC-based gene therapy, milder conditioning regimens, shock and kill strategies as well as other promising approaches, and thereby design a practical option for most patients with HIV for a functional cure.

## Author Contributions

JD and YuL authored and reviewed the manuscript. JD and YaL prepared tables. All authors contributed to manuscript revision, read, and approved the submitted version.

## Funding

The research was supported by the Xinglin Scholar Research Promotion Project of Chengdu University of TCM (grant number XSGG2020001). The funders had no role in study design, data collection and analysis, decision to publish, or preparation of the manuscript.

## Conflict of Interest

The authors declare that the research was conducted in the absence of any commercial or financial relationships that could be construed as a potential conflict of interest.

## References

[1] Organization WH. Hiv/Aids (2020). Available at: https://www.who.int/news-room/fact-sheets/detail/hiv-aids.

[2] Kwong PD Wyatt R Robinson J Sweet RW Sodroski J Hendrickson WA. Structure of an HIV gp120 Envelope Glycoprotein in Complex With the CD4 Receptor and a Neutralizing Human Antibody. Nature (1998) 393(6686):648–59. 10.1038/31405 PMC5629912 9641677

[3] Moore JP Trkola A Dragic T. Co-Receptors for HIV-1 Entry. Curr Opin Immunol (1997) 9(4):551–62. 10.1016/s0952-7915(97)80110-0 9287172

[4] Gorry PR Ancuta P. Coreceptors and HIV-1 Pathogenesis. Curr HIV/AIDS Rep (2011) 8(1):45–53. 10.1007/s11904-010-0069-x 21188555

[5] Dube K Willenberg L Dee L Sylla L Taylor J Roebuck C. Re-Examining the HIV ‘Functional Cure’ Oxymoron: Time for Precise Terminology? J Virus Erad (2020) 6(4):100017. 10.1016/j.jve.2020.100017 33251025 PMC7646673

[6] Hutter G Nowak D Mossner M Ganepola S Mussig A Allers K. Long-Term Control of HIV by CCR5 Delta32/Delta32 Stem-Cell Transplantation. N Engl J Med (2009) 360(7):692–8. 10.1056/NEJMoa0802905 19213682

[7] Gupta RK Abdul-Jawad S McCoy LE Mok HP Peppa D Salgado M. HIV-1 Remission Following CCR5Delta32/Delta32 Haematopoietic Stem-Cell Transplantation. Nature (2019) 568(7751):244–8. 10.1038/s41586-019-1027-4 PMC7275870 30836379

[8] Henrich TJ Hanhauser E Marty FM Sirignano MN Keating S Lee TH. Antiretroviral-Free HIV-1 Remission and Viral Rebound After Allogeneic Stem Cell Transplantation: Report of 2 Cases. Ann Intern Med (2014) 161(5):319–27. 10.7326/M14-1027 PMC4236912 25047577

[9] Cummins NW Rizza S Litzow MR Hua S Lee GQ Einkauf K. Extensive Virologic and Immunologic Characterization in an HIV-infected Individual Following Allogeneic Stem Cell Transplant and Analytic Cessation of Antiretroviral Therapy: A Case Study. PloS Med (2017) 14(11):e1002461. 10.1371/journal.pmed.1002461 29182633 PMC5705162

[10] Peterson CW Kiem HP. Lessons From London and Berlin: Designing A Scalable Gene Therapy Approach for HIV Cure. Cell Stem Cell (2019) 24(5):685–7. 10.1016/j.stem.2019.04.010 31051132

[11] Saez-Cirion A Bacchus C Hocqueloux L Avettand-Fenoel V Girault I Lecuroux C. Post-Treatment HIV-1 Controllers With a Long-Term Virological Remission After the Interruption of Early Initiated Antiretroviral Therapy ANRS Visconti Study. PloS Pathog (2013) 9(3):e1003211. 10.1371/journal.ppat.1003211 23516360 PMC3597518

[12] International HIVCS Pereyra F Jia X McLaren PJ Telenti A de Bakker PI. The Major Genetic Determinants of HIV-1 Control Affect HLA Class I Peptide Presentation. Science (2010) 330(6010):1551–7. 10.1126/science.1195271 PMC3235490 21051598

[13] Lai Y. Early Treatment During Primary Infection Holds the Key to a Functional Cure for HIV. Intervirology (2014) 57(1):52–3. 10.1159/000354800 24157414

[14] Ji H Lu P Liu B Qu X Wang Y Jiang Z. Zinc-Finger Nucleases Induced by HIV-1 Tat Excise HIV-1 From the Host Genome in Infected and Latently Infected Cells. Mol Ther Nucleic Acids (2018) 12:67–74. 10.1016/j.omtn.2018.04.014 30195798 PMC6023959

[15] Shi B Li J Shi X Jia W Wen Y Hu X. Talen-Mediated Knockout of CCR5 Confers Protection Against Infection of Human Immunodeficiency Virus. J Acquir Immune Defic Syndr (2017) 74(2):229–41. 10.1097/QAI.0000000000001190 27749600

[16] Dash PK Kaminski R Bella R Su H Mathews S Ahooyi TM. Sequential LASER ART and CRISPR Treatments Eliminate HIV-1 in a Subset of Infected Humanized Mice. Nat Commun (2019) 10(1):2753. 10.1038/s41467-019-10366-y 31266936 PMC6606613

[17] Perez EE Wang J Miller JC Jouvenot Y Kim KA Liu O. Establishment of HIV-1 Resistance in CD4+ T Cells by Genome Editing Using Zinc-Finger Nucleases. Nat Biotechnol (2008) 26(7):808–16. 10.1038/nbt1410 PMC3422503 18587387

[18] Holt N Wang J Kim K Friedman G Wang X Taupin V. Human Hematopoietic Stem/Progenitor Cells Modified by Zinc-Finger Nucleases Targeted to CCR5 Control HIV-1 In Vivo. Nat Biotechnol (2010) 28(8):839–47. 10.1038/nbt.1663 PMC3080757 20601939

[19] Li L Krymskaya L Wang J Henley J Rao A Cao LF. Genomic Editing of the HIV-1 Coreceptor CCR5 in Adult Hematopoietic Stem and Progenitor Cells Using Zinc Finger Nucleases. Mol Ther (2013) 21(6):1259–69. 10.1038/mt.2013.65 PMC3677314 23587921

[20] DiGiusto DL Cannon PM Holmes MC Li L Rao A Wang J. Preclinical Development and Qualification of ZFN-mediated CCR5 Disruption in Human Hematopoietic Stem/Progenitor Cells. Mol Ther Methods Clin Dev (2016) 3:16067. 10.1038/mtm.2016.67 27900346 PMC5102145

[21] Bach C Sherman W Pallis J Patra P Bajwa H. Evaluation of Novel Design Strategies for Developing Zinc Finger Nucleases Tools for Treating Human Diseases. Biotechnol Res Int (2014) 2014:970595. 10.1155/2014/970595 24808958 PMC3997970

[22] Cai Y Bak RO Mikkelsen JG. Targeted Genome Editing by Lentiviral Protein Transduction of Zinc-Finger and TAL-effector Nucleases. Elife (2014) 3:e01911. 10.7554/eLife.01911 24843011 PMC3996624

[23] Nalla AK Trobridge GD. Prospects for Foamy Viral Vector Anti-HIV Gene Therapy. Biomedicines (2016) 4(2):8. 10.3390/biomedicines4020008 PMC5344253 28536375

[24] Tebas P Stein D Tang WW Frank I Wang SQ Lee G. Gene Editing of CCR5 in Autologous CD4 T Cells of Persons Infected With HIV. N Engl J Med (2014) 370(10):901–10. 10.1056/NEJMoa1300662 PMC4084652 24597865

[25] Ricciardi AS Quijano E Putman R Saltzman WM Glazer PM. Peptide Nucleic Acids as a Tool for Site-Specific Gene Editing. Molecules (2018) 23(3):632. 10.3390/molecules23030632 PMC5946696 29534473

[26] Schleifman EB Bindra R Leif J del Campo J Rogers FA Uchil P. Targeted Disruption of the CCR5 Gene in Human Hematopoietic Stem Cells Stimulated by Peptide Nucleic Acids. Chem Biol (2011) 18(9):1189–98. 10.1016/j.chembiol.2011.07.010 PMC3183429 21944757

[27] Joung JK Sander JD. Talens: A Widely Applicable Technology for Targeted Genome Editing. Nat Rev Mol Cell Biol (2013) 14(1):49–55. 10.1038/nrm3486 23169466 PMC3547402

[28] Mussolino C Alzubi J Fine EJ Morbitzer R Cradick TJ Lahaye T. Talens Facilitate Targeted Genome Editing in Human Cells With High Specificity and Low Cytotoxicity. Nucleic Acids Res (2014) 42(10):6762–73. 10.1093/nar/gku305 PMC4041469 24792154

[29] Yu AQ Ding Y Lu ZY Hao YZ Teng ZP Yan SR. Talens-Mediated Homozygous CCR5Delta32 Mutations Endow CD4+ U87 Cells With Resistance Against HIV1 Infection. Mol Med Rep (2018) 17(1):243–9. 10.3892/mmr.2017.7889 PMC5780131 29115572

[30] Romito M Juillerat A Kok YL Hildenbeutel M Rhiel M Andrieux G. Preclinical Evaluation of a Novel Talen Targeting Ccr5 Confirms Efficacy and Safety in Conferring Resistance to HIV-1 Infection. Biotechnol J (2020) 16(1):e2000023. 10.1002/biot.202000023 33103367

[31] Allen AG Chung CH Atkins A Dampier W Khalili K Nonnemacher MR. Gene Editing of HIV-1 Co-Receptors to Prevent and/or Cure Virus Infection. Front Microbiol (2018) 9:2940. 10.3389/fmicb.2018.02940 30619107 PMC6304358

[32] Nerys-Junior A Braga-Dias LP Pezzuto P Cotta-de-Almeida V Tanuri A. Comparison of the Editing Patterns and Editing Efficiencies of TALEN and CRISPR-Cas9 When Targeting the Human CCR5 Gene. Genet Mol Biol (2018) 41(1):167–79. 10.1590/1678-4685-GMB-2017-0065 PMC5901495 29583154

[33] Teque F Ye L Xie F Wang J Morvan MG Kan YW. Genetically-Edited Induced Pluripotent Stem Cells Derived From HIV-1-infected Patients on Therapy can Give Rise to Immune Cells Resistant to HIV-1 Infection. AIDS (2020) 34(8):1141–9. 10.1097/QAD.0000000000002539 32287059

[34] Mandal PK Ferreira LM Collins R Meissner TB Boutwell CL Friesen M. Efficient Ablation of Genes in Human Hematopoietic Stem and Effector Cells Using CRISPR/Cas9. Cell Stem Cell (2014) 15(5):643–52. 10.1016/j.stem.2014.10.004 PMC4269831 25517468

[35] Xu L Yang H Gao Y Chen Z Xie L Liu Y. Crispr/Cas9-Mediated CCR5 Ablation in Human Hematopoietic Stem/Progenitor Cells Confers Hiv-1 Resistance In Vivo. Mol Ther (2017) 25(8):1782–9. 10.1016/j.ymthe.2017.04.027 PMC5542791 28527722

[36] Schmidt JK Strelchenko N Park MA Kim YH Mean KD Schotzko ML. Genome Editing of CCR5 by CRISPR-Cas9 in Mauritian Cynomolgus Macaque Embryos. Sci Rep (2020) 10(1):18457. 10.1038/s41598-020-75295-z 33116147 PMC7595107

[37] Yu S Ou Y Xiao H Li J Adah D Liu S. Experimental Treatment of SIV-Infected Macaques Via Autograft of CCR5-Disrupted Hematopoietic Stem and Progenitor Cells. Mol Ther Methods Clin Dev (2020) 17:520–31. 10.1016/j.omtm.2020.03.004 PMC7114624 32258215

[38] Ran FA Cong L Yan WX Scott DA Gootenberg JS Kriz AJ. In Vivo Genome Editing Using Staphylococcus Aureus Cas9. Nature (2015) 520(7546):186–91. 10.1038/nature14299 PMC4393360 25830891

[39] Xiao Q Chen S Wang Q Liu Z Liu S Deng H. CCR5 Editing by Staphylococcus Aureus Cas9 in Human Primary CD4(+) T Cells and Hematopoietic Stem/Progenitor Cells Promotes HIV-1 Resistance and CD4(+) T Cell Enrichment in Humanized Mice. Retrovirology (2019) 16(1):15. 10.1186/s12977-019-0477-y 31186067 PMC6560749

[40] Xu L Wang J Liu Y Xie L Su B Mou D. Crispr-Edited Stem Cells in a Patient With HIV and Acute Lymphocytic Leukemia. N Engl J Med (2019) 381(13):1240–7. 10.1056/NEJMoa1817426 31509667

[41] O’Brien TR Winkler C Dean M Nelson JA Carrington M Michael NL. HIV-1 Infection in a Man Homozygous for CCR5 Delta 32. Lancet (1997) 349(9060):1219. 10.1016/s0140-6736(97)24017-1 9130945

[42] Sheppard HW Celum C Michael NL O’Brien S Dean M Carrington M. HIV-1 Infection in Individuals With the CCR5-Delta32/Delta32 Genotype: Acquisition of Syncytium-Inducing Virus At Seroconversion. J Acquir Immune Defic Syndr (2002) 29(3):307–13. 10.1097/00126334-200203010-00013 11873082

[43] Henrich TJ Hanhauser E Hu Z Stellbrink HJ Noah C Martin JN. Viremic Control and Viral Coreceptor Usage in Two HIV-1-infected Persons Homozygous for CCR5 Delta32. AIDS (2015) 29(8):867–76. 10.1097/QAD.0000000000000629 PMC4473772 25730507

[44] Smolen-Dzirba J Rosinska M Janiec J Beniowski M Cycon M Bratosiewicz-Wasik J. Hiv-1 Infection in Persons Homozygous for CCR5-Delta32 Allele: The Next Case and the Review. AIDS Rev (2017) 19(4):219–30. 28534889

[45] Wilen CB Wang J Tilton JC Miller JC Kim KA Rebar EJ. Engineering HIV-resistant Human CD4+ T Cells With CXCR4-specific Zinc-Finger Nucleases. PloS Pathog (2011) 7(4):e1002020. 10.1371/journal.ppat.1002020 21533216 PMC3077364

[46] Yuan J Wang J Crain K Fearns C Kim KA Hua KL. Zinc-Finger Nuclease Editing of Human Cxcr4 Promotes HIV-1 Cd4(+) T Cell Resistance and Enrichment. Mol Ther (2012) 20(4):849–59. 10.1038/mt.2011.310 PMC3321595 22273578

[47] Wang Q Chen S Xiao Q Liu Z Liu S Hou P. Genome Modification of CXCR4 by Staphylococcus Aureus Cas9 Renders Cells Resistance to HIV-1 Infection. Retrovirology (2017) 14(1):51. 10.1186/s12977-017-0375-0 29141633 PMC5688617

[48] Didigu CA Wilen CB Wang J Duong J Secreto AJ Danet-Desnoyers GA. Simultaneous Zinc-Finger Nuclease Editing of the HIV Coreceptors Ccr5 and Cxcr4 Protects CD4+ T Cells From HIV-1 Infection. Blood (2014) 123(1):61–9. 10.1182/blood-2013-08-521229 PMC3879906 24162716

[49] Yu S Yao Y Xiao H Li J Liu Q Yang Y. Simultaneous Knockout of CXCR4 and CCR5 Genes in CD4+ T Cells Via CRISPR/Cas9 Confers Resistance to Both X4- and R5-Tropic Human Immunodeficiency Virus Type 1 Infection. Hum Gene Ther (2018) 29(1):51–67. 10.1089/hum.2017.032 28599597

[50] Hou P Chen S Wang S Yu X Chen Y Jiang M. Genome Editing of CXCR4 by CRISPR/cas9 Confers Cells Resistant to HIV-1 Infection. Sci Rep (2015) 5:15577. 10.1038/srep15577 26481100 PMC4612538

[51] Liu Z Chen S Jin X Wang Q Yang K Li C. Genome Editing of the HIV Co-Receptors CCR5 and CXCR4 by CRISPR-Cas9 Protects CD4(+) T Cells From HIV-1 Infection. Cell Biosci (2017) 7:47. 10.1186/s13578-017-0174-2 28904745 PMC5591563

[52] Ma Q Jones D Borghesani PR Segal RA Nagasawa T Kishimoto T. Impaired B-lymphopoiesis, Myelopoiesis, and Derailed Cerebellar Neuron Migration in CXCR4- and SDF-1-deficient Mice. Proc Natl Acad Sci USA (1998) 95(16):9448–53. 10.1073/pnas.95.16.9448 PMC21358 9689100

[53] Dar A Kollet O Lapidot T. Mutual, Reciprocal SDF-1/CXCR4 Interactions Between Hematopoietic and Bone Marrow Stromal Cells Regulate Human Stem Cell Migration and Development in NOD/SCID Chimeric Mice. Exp Hematol (2006) 34(8):967–75. 10.1016/j.exphem.2006.04.002 16863903

[54] Liu S Wang Q Yu X Li Y Guo Y Liu Z. HIV-1 Inhibition in Cells With CXCR4 Mutant Genome Created by CRISPR-Cas9 and piggyBac Recombinant Technologies. Sci Rep (2018) 8(1):8573. 10.1038/s41598-018-26894-4 29872154 PMC5988798

[55] Liu Y Zhou J Pan JA Mabiala P Guo D. A Novel Approach to Block HIV-1 Coreceptor CXCR4 in non-Toxic Manner. Mol Biotechnol (2014) 56(10):890–902. 10.1007/s12033-014-9768-7 24845753

[56] Cadima-Couto I Tauzin A Freire JM Figueira TN Silva RDM Perez-Peinado C. Anti-HIV-1 Activity of pepRF1, a Proteolysis-Resistant Cxcr4 Antagonist Derived From Dengue Virus Capsid Protein. ACS Infect Dis (2021) 7(1):6–22. 10.1021/acsinfecdis.9b00507 33319557

[57] Kumar Bhardwaj V Purohit R Kumar S. Himalayan Bioactive Molecules as Potential Entry Inhibitors for the Human Immunodeficiency Virus. Food Chem (2020) 347:128932. 10.1016/j.foodchem.2020.128932 33465692

[58] Burt RK Marmont A Oyama Y Slavin S Arnold R Hiepe F. Randomized Controlled Trials of Autologous Hematopoietic Stem Cell Transplantation for Autoimmune Diseases: The Evolution From Myeloablative to Lymphoablative Transplant Regimens. Arthritis Rheum (2006) 54(12):3750–60. 10.1002/art.22256 17133541

[59] Orozco JJ Kenoyer A Balkin ER Gooley TA Hamlin DK Wilbur DS. Anti-CD45 Radioimmunotherapy Without TBI Before Transplantation Facilitates Persistent Haploidentical Donor Engraftment. Blood (2016) 127(3):352–9. 10.1182/blood-2014-12-617019 PMC4722286 26576864

[60] Cassaday RD Press OW Pagel JM Rajendran JG Gooley TA Fisher DR. Phase I Study of a CD45-Targeted Antibody-Radionuclide Conjugate for High-Risk Lymphoma. Clin Cancer Res (2019) 25(23):6932–8. 10.1158/1078-0432.CCR-19-1567 PMC6891166 31481510

[61] Bethge WA Wilbur DS Sandmaier BM. Radioimmunotherapy as non-Myeloablative Conditioning for Allogeneic Marrow Transplantation. Leuk Lymphoma (2006) 47(7):1205–14. 10.1080/00423110500485822 16923548

[62] Canakci M Singh K Munkhbat O Shanthalingam S Mitra A Gordon M. Targeting CD4(+) Cells With Anti-CD4 Conjugated Mertansine-Loaded Nanogels. Biomacromolecules (2020) 21(6):2473–81. 10.1021/acs.biomac.0c00442 PMC7354862 32383874

[63] Palchaudhuri R Saez B Hoggatt J Schajnovitz A Sykes DB Tate TA. Non-Genotoxic Conditioning for Hematopoietic Stem Cell Transplantation Using a Hematopoietic-Cell-Specific Internalizing Immunotoxin. Nat Biotechnol (2016) 34(7):738–45. 10.1038/nbt.3584 PMC5179034 27272386

[64] Czechowicz A Palchaudhuri R Scheck A Hu Y Hoggatt J Saez B. Selective Hematopoietic Stem Cell Ablation Using CD117-antibody-drug-conjugates Enables Safe and Effective Transplantation With Immunity Preservation. Nat Commun (2019) 10(1):617. 10.1038/s41467-018-08201-x 30728354 PMC6365495

[65] Sogaard OS Graversen ME Leth S Olesen R Brinkmann CR Nissen SK. The Depsipeptide Romidepsin Reverses Hiv-1 Latency In Vivo. PloS Pathog (2015) 11(9):e1005142. 10.1371/journal.ppat.1005142 26379282 PMC4575032

[66] Leth S Schleimann MH Nissen SK Hojen JF Olesen R Graversen ME. Combined Effect of Vacc-4x, Recombinant Human Granulocyte Macrophage Colony-Stimulating Factor Vaccination, and Romidepsin on the HIV-1 Reservoir (REDUC): A Single-Arm, Phase 1B/2A Trial. Lancet HIV (2016) 3(10):e463–72. 10.1016/S2352-3018(16)30055-8 27658863

[67] McMahon DK Zheng L Cyktor JC Aga E Macatangay BJ Godfrey C. A Phase I/II Randomized, Placebo-Controlled Trial of Romidepsin in Persons With HIV-1 on Suppressive Antiretroviral Therapy to Assess Safety and Activation of HIV-1 Expression (A5315). J Infect Dis (2020). 10.1093/infdis/jiaa777 PMC8366434 34398236

[68] Archin NM Liberty AL Kashuba AD Choudhary SK Kuruc JD Crooks AM. Administration of Vorinostat Disrupts HIV-1 Latency in Patients on Antiretroviral Therapy. Nature (2012) 487(7408):482–5. 10.1038/nature11286 PMC3704185 22837004

[69] Garrido C Tolstrup M Sogaard OS Rasmussen TA Allard B Soriano-Sarabia N. In-Vivo Administration of Histone Deacetylase Inhibitors Does Not Impair Natural Killer Cell Function in HIV+ Individuals. AIDS (2019) 33(4):605–13. 10.1097/QAD.0000000000002112 PMC6400302 30830886

[70] Archin NM Kirchherr JL Sung JA Clutton G Sholtis K Xu Y. Interval Dosing With the HDAC Inhibitor Vorinostat Effectively Reverses HIV Latency. J Clin Invest (2017) 127(8):3126–35. 10.1172/JCI92684 PMC5531421 28714868

[71] Archin NM Bateson R Tripathy MK Crooks AM Yang KH Dahl NP. HIV-1 Expression Within Resting CD4+ T Cells After Multiple Doses of Vorinostat. J Infect Dis (2014) 210(5):728–35. 10.1093/infdis/jiu155 PMC4148603 24620025

[72] Mota TM Rasmussen TA Rhodes A Tennakoon S Dantanarayana A Wightman F. No Adverse Safety or Virological Changes 2 Years Following Vorinostat in HIV-infected Individuals on Antiretroviral Therapy. AIDS (2017) 31(8):1137–41. 10.1097/QAD.0000000000001442 PMC5496768 28301423

[73] Elliott JH Wightman F Solomon A Ghneim K Ahlers J Cameron MJ. Activation of HIV Transcription With Short-Course Vorinostat in HIV-infected Patients on Suppressive Antiretroviral Therapy. PloS Pathog (2014) 10(10):e1004473. 10.1371/journal.ppat.1004473 25393648 PMC4231123

[74] Day S Mathews A Blumberg M Vu T Rennie S Tucker JD. Broadening Community Engagement in Clinical Research: Designing and Assessing a Pilot Crowdsourcing Project to Obtain Community Feedback on an HIV Clinical Trial. Clin Trials (2020) 17(3):306–13. 10.1177/1740774520902741 PMC7255944 32009466

[75] Fidler S Stohr W Pace M Dorrell L Lever A Pett S. Antiretroviral Therapy Alone Versus Antiretroviral Therapy With a Kick and Kill Approach, on Measures of the HIV Reservoir in Participants With Recent HIV Infection (the RIVER Trial): A Phase 2, Randomised Trial. Lancet (2020) 395(10227):888–98. 10.1016/S0140-6736(19)32990-3 32085823

[76] Rasmussen TA Tolstrup M Brinkmann CR Olesen R Erikstrup C Solomon A. Panobinostat, a Histone Deacetylase Inhibitor, for Latent-Virus Reactivation in HIV-infected Patients on Suppressive Antiretroviral Therapy: A Phase 1/2, Single Group, Clinical Trial. Lancet HIV (2014) 1(1):e13–21. 10.1016/S2352-3018(14)70014-1 26423811

[77] Curreli F Ahmed S Victor SMB Debnath AK. Identification of Combinations of Protein Kinase C Activators and Histone Deacetylase Inhibitors That Potently Reactivate Latent Hiv. Viruses (2020) 12(6):609. 10.3390/v12060609 PMC7354613 32503121

[78] Mota TM McCann CD Danesh A Huang SH Magat DB Ren Y. Integrated Assessment of Viral Transcription, Antigen Presentation, and CD8(+) T Cell Function Reveals Multiple Limitations of Class I-Selective Histone Deacetylase Inhibitors During HIV-1 Latency Reversal. J Virol (2020) 94(9):e01845–19. 10.1128/JVI.01845-19 PMC7163115 32051267

[79] Samer S Arif MS Giron LB Zukurov JPL Hunter J Santillo BT. Nicotinamide Activates Latent HIV-1 Ex Vivo in ART Suppressed Individuals, Revealing Higher Potency Than the Association of Two Methyltransferase Inhibitors, Chaetocin and BIX01294. Braz J Infect Dis (2020) 24(2):150–9. 10.1016/j.bjid.2020.01.005 PMC9392037 32105620

[80] Cary DC Peterlin BM. Procyanidin Trimer C1 Reactivates Latent HIV as a Triple Combination Therapy With Kansui and JQ1. PloS One (2018) 13(11):e0208055. 10.1371/journal.pone.0208055 30475902 PMC6258234

[81] Li G Zhang Z Reszka-Blanco N Li F Chi L Ma J. Specific Activation In Vivo of HIV-1 by a Bromodomain Inhibitor From Monocytic Cells in Humanized Mice Under Antiretroviral Therapy. J Virol (2019) 93(12):e00233–19. 10.1128/JVI.00233-19 PMC6613761 30971469

[82] Boehm D Calvanese V Dar RD Xing S Schroeder S Martins L. BET Bromodomain-Targeting Compounds Reactivate HIV From Latency Via a Tat-independent Mechanism. Cell Cycle (2013) 12(3):452–62. 10.4161/cc.23309 PMC3587446 23255218

[83] Huang H Liu S Jean M Simpson S Huang H Merkley M. A Novel Bromodomain Inhibitor Reverses HIV-1 Latency Through Specific Binding With BRD4 to Promote Tat and P-TEFb Association. Front Microbiol (2017) 8:1035. 10.3389/fmicb.2017.01035 28638377 PMC5461361

[84] Lu P Qu X Shen Y Jiang Z Wang P Zeng H. The BET Inhibitor OTX015 Reactivates Latent HIV-1 Through P-Tefb. Sci Rep (2016) 6:24100. 10.1038/srep24100 27067814 PMC4828723

[85] Liang T Zhang X Lai F Lin J Zhou C Xu X. A Novel Bromodomain Inhibitor, CPI-203, Serves as an HIV-1 Latency-Reversing Agent by Activating Positive Transcription Elongation Factor B. Biochem Pharmacol (2019) 164:237–51. 10.1016/j.bcp.2019.04.005 30991051

[86] Lu P Shen Y Yang H Wang Y Jiang Z Yang X. BET Inhibitors RVX-208 and PFI-1 Reactivate HIV-1 From Latency. Sci Rep (2017) 7(1):16646. 10.1038/s41598-017-16816-1 29192216 PMC5709369

[87] Gohda J Suzuki K Liu K Xie X Takeuchi H Inoue JI. Bi-2536 and BI-6727, Dual Polo-like Kinase/Bromodomain Inhibitors, Effectively Reactivate Latent HIV-1. Sci Rep (2018) 8(1):3521. 10.1038/s41598-018-21942-5 29476067 PMC5824842

[88] Zhang XX Lin J Liang TZ Duan H Tan XH Xi BM. The BET Bromodomain Inhibitor Apabetalone Induces Apoptosis of Latent HIV-1 Reservoir Cells Following Viral Reactivation. Acta Pharmacol Sin (2019) 40(1):98–110. 10.1038/s41401-018-0027-5 29789664 PMC6318340

[89] Nguyen K Das B Dobrowolski C Karn J. Multiple Histone Lysine Methyltransferases are Required for the Establishment and Maintenance of HIV-1 Latency. mBio (2017) 8(1):e00133–17. 10.1128/mBio.00133-17 PMC5347344 28246360

[90] Turner AW Dronamraju R Potjewyd F James KS Winecoff DK Kirchherr JL. Evaluation of EED Inhibitors as a Class of PRC2-Targeted Small Molecules for HIV Latency Reversal. ACS Infect Dis (2020) 6(7):1719–33. 10.1021/acsinfecdis.9b00514 PMC7359025 32347704

[91] Sharma AL Hokello J Sonti S Zicari S Sun L Alqatawni A. Cbf-1 Promotes the Establishment and Maintenance of HIV Latency by Recruiting Polycomb Repressive Complexes, PRC1 and PRC2, At HIV Ltr. Viruses (2020) 12(9):1040. 10.3390/v12091040 PMC7551090 32961937

[92] Friedman J Cho WK Chu CK Keedy KS Archin NM Margolis DM. Epigenetic Silencing of HIV-1 by the Histone H3 Lysine 27 Methyltransferase Enhancer of Zeste 2. J Virol (2011) 85(17):9078–89. 10.1128/JVI.00836-11 PMC3165831 21715480

[93] Bouchat S Delacourt N Kula A Darcis G Van Driessche B Corazza F. Sequential Treatment With 5-Aza-2’-Deoxycytidine and Deacetylase Inhibitors Reactivates HIV-1. EMBO Mol Med (2016) 8(2):117–38. 10.15252/emmm.201505557 PMC4734845 26681773

[94] Yang H Li X Yang X Lu P Wang Y Jiang Z. Dual Effects of the Novel Ingenol Derivatives on the Acute and Latent HIV-1 Infections. Antiviral Res (2019) 169:104555. 10.1016/j.antiviral.2019.104555 31295520

[95] Wang P Lu P Qu X Shen Y Zeng H Zhu X. Reactivation of HIV-1 From Latency by an Ingenol Derivative From Euphorbia Kansui. Sci Rep (2017) 7(1):9451. 10.1038/s41598-017-07157-0 28842560 PMC5573388

[96] Liu Q Li W Huang L Asada Y Morris-Natschke SL Chen CH. Identification, Structural Modification, and Dichotomous Effects on Human Immunodeficiency Virus Type 1 (HIV-1) Replication of Ingenane Esters From Euphorbia Kansui. Eur J Med Chem (2018) 156:618–27. 10.1016/j.ejmech.2018.07.020 PMC6217806 30031972

[97] Cary DC Fujinaga K Peterlin BM. Euphorbia Kansui Reactivates Latent Hiv. PloS One (2016) 11(12):e0168027. 10.1371/journal.pone.0168027 27977742 PMC5158021

[98] Gutierrez C Serrano-Villar S Madrid-Elena N Perez-Elias MJ Martin ME Barbas C. Bryostatin-1 for Latent Virus Reactivation in HIV-infected Patients on Antiretroviral Therapy. AIDS (2016) 30(9):1385–92. 10.1097/QAD.0000000000001064 26891037

[99] Marsden MD Kovochich M Suree N Shimizu S Mehta R Cortado R. HIV Latency in the Humanized BLT Mouse. J Virol (2012) 86(1):339–47. 10.1128/JVI.06366-11 PMC3255908 22072769

[100] Geng G Liu B Chen C Wu K Liu J Zhang Y. Development of an Attenuated Tat Protein as a Highly-effective Agent to Specifically Activate HIV-1 Latency. Mol Ther (2016) 24(9):1528–37. 10.1038/mt.2016.117 PMC5113098 27434587

[101] Novis CL Archin NM Buzon MJ Verdin E Round JL Lichterfeld M. Reactivation of Latent HIV-1 in Central Memory CD4(+) T Cells Through TLR-1/2 Stimulation. Retrovirology (2013) 10:119. 10.1186/1742-4690-10-119 24156240 PMC3826617

[102] Riddler SA Para M Benson CA Mills A Ramgopal M DeJesus E. Vesatolimod, a Toll-Like Receptor 7 Agonist, Induces Immune Activation in Virally Suppressed Adults With HIV-1. Clin Infect Dis (2020). 10.1093/cid/ciaa1534 33043969

[103] Lim SY Osuna CE Hraber PT Hesselgesser J Gerold JM Barnes TL. TLR7 Agonists Induce Transient Viremia and Reduce the Viral Reservoir in SIV-infected Rhesus Macaques on Antiretroviral Therapy. Sci Transl Med (2018) 10(439):eaao4521. 10.1126/scitranslmed.aao4521 29720451 PMC5973480

[104] Schlaepfer E Speck RF. TLR8 Activates HIV From Latently Infected Cells of Myeloid-Monocytic Origin Directly Via the MAPK Pathway and From Latently Infected CD4+ T Cells Indirectly Via TNF-Alpha. J Immunol (2011) 186(7):4314–24. 10.4049/jimmunol.1003174 21357269

[105] Vibholm LK Konrad CV Schleimann MH Frattari G Winckelmann A Klastrup V. Effects of 24-Week Toll-like Receptor 9 Agonist Treatment in HIV Type 1+ Individuals. AIDS (2019) 33(8):1315–25. 10.1097/QAD.0000000000002213 30932955

[106] Vibholm L Schleimann MH Hojen JF Benfield T Offersen R Rasmussen K. Short-Course Toll-Like Receptor 9 Agonist Treatment Impacts Innate Immunity and Plasma Viremia in Individuals With Human Immunodeficiency Virus Infection. Clin Infect Dis (2017) 64(12):1686–95. 10.1093/cid/cix201 PMC5849129 28329286

[107] Winckelmann AA Munk-Petersen LV Rasmussen TA Melchjorsen J Hjelholt TJ Montefiori D. Administration of a Toll-like Receptor 9 Agonist Decreases the Proviral Reservoir in Virologically Suppressed HIV-infected Patients. PloS One (2013) 8(4):e62074. 10.1371/journal.pone.0062074 23637967 PMC3637371

[108] Evans VA van der Sluis RM Solomon A Dantanarayana A McNeil C Garsia R. Programmed Cell Death-1 Contributes to the Establishment and Maintenance of HIV-1 Latency. AIDS (2018) 32(11):1491–7. 10.1097/QAD.0000000000001849 PMC6026054 29746296

[109] Wightman F Solomon A Kumar SS Urriola N Gallagher K Hiener B. Effect of Ipilimumab on the HIV Reservoir in an HIV-infected Individual With Metastatic Melanoma. AIDS (2015) 29(4):504–6. 10.1097/QAD.0000000000000562 PMC4492799 25628259

[110] Gay CL Bosch RJ Ritz J Hataye JM Aga E Tressler RL. Clinical Trial of the Anti-PD-L1 Antibody BMS-936559 in HIV-1 Infected Participants on Suppressive Antiretroviral Therapy. J Infect Dis (2017) 215(11):1725–33. 10.1093/infdis/jix191 PMC5790148 28431010

[111] Sanz M Madrid-Elena N Serrano-Villar S Vallejo A Gutierrez C Moreno S. Effect of the Use of Galectin-9 and Blockade of TIM-3 Receptor in the Latent Cellular Reservoir of HIV-1. J Virol (2020) 95(5):e02214–20. 10.1128/JVI.02214-20 PMC8092815 33361434

[112] Madrid-Elena N Garcia-Bermejo ML Serrano-Villar S Diaz-de Santiago A Sastre B Gutierrez C. Maraviroc Is Associated With Latent Hiv-1 Reactivation Through NF-kappaB Activation in Resting Cd4(+) T Cells From HIV-Infected Individuals on Suppressive Antiretroviral Therapy. J Virol (2018) 92(9):e01931–17. 10.1128/JVI.01931-17 PMC5899181 29444937

[113] Gutierrez C Diaz L Vallejo A Hernandez-Novoa B Abad M Madrid N. Intensification of Antiretroviral Therapy With a CCR5 Antagonist in Patients With Chronic HIV-1 Infection: Effect on T Cells Latently Infected. PloS One (2011) 6(12):e27864. 10.1371/journal.pone.0027864 22174752 PMC3234247

[114] McBrien JB Mavigner M Franchitti L Smith SA White E Tharp GK. Robust and Persistent Reactivation of SIV and HIV by N-803 and Depletion of CD8(+) Cells. Nature (2020) 578(7793):154–9. 10.1038/s41586-020-1946-0 PMC7580846 31969705

[115] Nixon CC Mavigner M Sampey GC Brooks AD Spagnuolo RA Irlbeck DM. Systemic HIV and SIV Latency Reversal Via non-Canonical NF-kappaB Signalling In Vivo. Nature (2020) 578(7793):160–5. 10.1038/s41586-020-1951-3 PMC7111210 31969707

[116] Pache L Marsden MD Teriete P Portillo AJ Heimann D Kim JT. Pharmacological Activation of Non-canonical NF-Kappab Signaling Activates Latent Hiv-1 Reservoirs In Vivo. Cell Rep Med (2020) 1(3):100037. 10.1016/j.xcrm.2020.100037 33205060 PMC7659604

[117] Elliott JH McMahon JH Chang CC Lee SA Hartogensis W Bumpus N. Short-Term Administration of Disulfiram for Reversal of Latent HIV Infection: A Phase 2 Dose-Escalation Study. Lancet HIV (2015) 2(12):e520–9. 10.1016/S2352-3018(15)00226-X PMC5108570 26614966

[118] Ylisastigui L Archin NM Lehrman G Bosch RJ Margolis DM. Coaxing HIV-1 From Resting CD4 T Cells: Histone Deacetylase Inhibition Allows Latent Viral Expression. AIDS (2004) 18(8):1101–8. 10.1097/00002030-200405210-00003 15166525

[119] Rasmussen TA Schmeltz Sogaard O Brinkmann C Wightman F Lewin SR Melchjorsen J. Comparison of HDAC Inhibitors in Clinical Development: Effect on HIV Production in Latently Infected Cells and T-cell Activation. Hum Vaccin Immunother (2013) 9(5):993–1001. 10.4161/hv.23800 23370291 PMC3899169

[120] Zhao M De Crignis E Rokx C Verbon A van Gelder T Mahmoudi T. T Cell Toxicity of HIV Latency Reversing Agents. Pharmacol Res (2019) 139:524–34. 10.1016/j.phrs.2018.10.023 30366100

[121] Norton NJ Mok HP Sharif F Hirst JC Lever AML. Hiv Silencing and Inducibility are Heterogeneous and Are Affected by Factors Intrinsic to the Virus. mBio (2019) 10(3):e0188–19. 10.1128/mBio.00188-19 PMC6593397 31239371

[122] Banerjee C Archin N Michaels D Belkina AC Denis GV Bradner J. BET Bromodomain Inhibition as a Novel Strategy for Reactivation of HIV-1. J Leukoc Biol (2012) 92(6):1147–54. 10.1189/jlb.0312165 PMC3501896 22802445

[123] Qi W Zhao K Gu J Huang Y Wang Y Zhang H. An Allosteric PRC2 Inhibitor Targeting the H3K27me3 Binding Pocket of EED. Nat Chem Biol (2017) 13(4):381–8. 10.1038/nchembio.2304 28135235

[124] Jiang G Dandekar S. Targeting NF-kappaB Signaling With Protein Kinase C Agonists as an Emerging Strategy for Combating HIV Latency. AIDS Res Hum Retroviruses (2015) 31(1):4–12. 10.1089/AID.2014.0199 25287643 PMC4287114

[125] Jiang G Mendes EA Kaiser P Sankaran-Walters S Tang Y Weber MG. Reactivation of HIV Latency by a Newly Modified Ingenol Derivative Via Protein Kinase Cdelta-NF-kappaB Signaling. AIDS (2014) 28(11):1555–66. 10.1097/QAD.0000000000000289 PMC4922310 24804860

[126] Bullen CK Laird GM Durand CM Siliciano JD Siliciano RF. New Ex Vivo Approaches Distinguish Effective and Ineffective Single Agents for Reversing HIV-1 Latency In Vivo. Nat Med (2014) 20(4):425–9. 10.1038/nm.3489 PMC3981911 24658076

[127] Beans EJ Fournogerakis D Gauntlett C Heumann LV Kramer R Marsden MD. Highly Potent, Synthetically Accessible Prostratin Analogs Induce Latent HIV Expression In Vitro and Ex Vivo. Proc Natl Acad Sci USA (2013) 110(29):11698–703. 10.1073/pnas.1302634110 PMC3718093 23812750

[128] Desimio MG Giuliani E Ferraro AS Adorno G Doria M. In Vitro Exposure to Prostratin But Not Bryostatin-1 Improves Natural Killer Cell Functions Including Killing of CD4(+) T Cells Harboring Reactivated Human Immunodeficiency Virus. Front Immunol (2018) 9:1514. 10.3389/fimmu.2018.01514 30008723 PMC6033996

[129] Pavlick AC Wu J Roberts J Rosenthal MA Hamilton A Wadler S. Phase I Study of Bryostatin 1, a Protein Kinase C Modulator, Preceding Cisplatin in Patients With Refractory non-Hematologic Tumors. Cancer Chemother Pharmacol (2009) 64(4):803–10. 10.1007/s00280-009-0931-y PMC3901370 19221754

[130] Dental C Proust A Ouellet M Barat C Tremblay MJ. Hiv-1 Latency-Reversing Agents Prostratin and Bryostatin-1 Induce Blood-Brain Barrier Disruption/Inflammation and Modulate Leukocyte Adhesion/Transmigration. J Immunol (2017) 198(3):1229–41. 10.4049/jimmunol.1600742 27994072

[131] Grau-Exposito J Luque-Ballesteros L Navarro J Curran A Burgos J Ribera E. Latency Reversal Agents Affect Differently the Latent Reservoir Present in Distinct CD4+ T Subpopulations. PloS Pathog (2019) 15(8):e1007991. 10.1371/journal.ppat.1007991 31425551 PMC6715238

[132] Taylor JP Armitage LH Aldridge DL Cash MN Wallet MA. Harmine Enhances the Activity of the HIV-1 Latency-Reversing Agents Ingenol A and SAHA. Biol Open (2020) 9(12):bio052969. 10.1242/bio.052969 33234703 PMC7774897

[133] Takeda K Kaisho T Akira S. Toll-Like Receptors. Annu Rev Immunol (2003) 21:335–76. 10.1146/annurev.immunol.21.120601.141126 12524386

[134] Schlaepfer E Audige A Joller H Speck RF. TLR7/8 Triggering Exerts Opposing Effects in Acute Versus Latent HIV Infection. J Immunol (2006) 176(5):2888–95. 10.4049/jimmunol.176.5.2888 16493046

[135] Offersen R Nissen SK Rasmussen TA Ostergaard L Denton PW Sogaard OS. A Novel Toll-Like Receptor 9 Agonist, Mgn1703, Enhances Hiv-1 Transcription and NK Cell-Mediated Inhibition of HIV-1-Infected Autologous Cd4+ T Cells. J Virol (2016) 90(9):4441–53. 10.1128/JVI.00222-16 PMC4836316 26889036

[136] Tsai A Irrinki A Kaur J Cihlar T Kukolj G Sloan DD. Toll-Like Receptor 7 Agonist Gs-9620 Induces Hiv Expression and HIV-Specific Immunity in Cells From HIV-Infected Individuals on Suppressive Antiretroviral Therapy. J Virol (2017) 91(8):e02166–16. 10.1128/JVI.02166-16 PMC5375698 28179531

[137] Ram RR Duatschek P Margot N Abram M Geleziunas R Hesselgesser J. Activation of HIV-specific Cd8(+) T-cells From HIV+ Donors by Vesatolimod. Antivir Ther (2020) 25(3):163–9. 10.3851/IMP3359 32420906

[138] Fromentin R Bakeman W Lawani MB Khoury G Hartogensis W DaFonseca S. Cd4+ T Cells Expressing Pd-1, TIGIT and LAG-3 Contribute to HIV Persistence During ART. PloS Pathog (2016) 12(7):e1005761. 10.1371/journal.ppat.1005761 27415008 PMC4944956

[139] McGary CS Deleage C Harper J Micci L Ribeiro SP Paganini S. Ctla-4(+)Pd-1(-) Memory Cd4(+) T Cells Critically Contribute to Viral Persistence in Antiretroviral Therapy-Suppressed, SIV-Infected Rhesus Macaques. Immunity (2017) 47(4):776–88.e5. 10.1016/j.immuni.2017.09.018 29045906 PMC5679306

[140] Van der Sluis RM Kumar NA Pascoe RD Zerbato JM Evans VA Dantanarayana AI. Combination Immune Checkpoint Blockade to Reverse Hiv Latency. J Immunol (2020) 204(5):1242–54. 10.4049/jimmunol.1901191 PMC7354848 31988180

[141] Rasmussen TA Rajdev L Rhodes A Dantanarayana A Tennakoon S Chea S. Impact of anti-PD-1 and anti-CTLA-4 on the HIV Reservoir in People Living With HIV With Cancer on Antiretroviral Therapy: The Aids Malignancy Consortium-095 Study. Clin Infect Dis (2021). 10.1093/cid/ciaa1530 PMC8492152 33677480

[142] Hong X Schouest B Xu H. Effects of Exosome on the Activation of CD4+ T Cells in Rhesus Macaques: A Potential Application for HIV Latency Reactivation. Sci Rep (2017) 7(1):15611. 10.1038/s41598-017-15961-x 29142313 PMC5688118

[143] Bosque A Famiglietti M Weyrich AS Goulston C Planelles V. Homeostatic Proliferation Fails to Efficiently Reactivate HIV-1 Latently Infected Central Memory CD4+ T Cells. PloS Pathog (2011) 7(10):e1002288. 10.1371/journal.ppat.1002288 21998586 PMC3188522

[144] McBrien JB Wong AKH White E Carnathan DG Lee JH Safrit JT. Combination of CD8beta Depletion and Interleukin-15 Superagonist N-803 Induces Virus Reactivation in Simian-Human Immunodeficiency Virus-Infected, Long-Term ART-Treated Rhesus Macaques. J Virol (2020) 94(19):e00755–20. 10.1128/JVI.00755-20 PMC7495383 32669328

[145] Webb GM Molden J Busman-Sahay K Abdulhaqq S Wu HL Weber WC. The Human IL-15 Superagonist N-803 Promotes Migration of Virus-Specific CD8+ T and NK Cells to B Cell Follicles But Does Not Reverse Latency in ART-suppressed, SHIV-Infected Macaques. PloS Pathog (2020) 16(3):e1008339. 10.1371/journal.ppat.1008339 32163523 PMC7093032

[146] Margolis DM Garcia JV. Countering HIV - Three’s the Charm? N Engl J Med (2018) 378(3):295–7. 10.1056/NEJMcibr1712494 29342376

[147] Xu L Pegu A Rao E Doria-Rose N Beninga J McKee K. Trispecific Broadly Neutralizing HIV Antibodies Mediate Potent SHIV Protection in Macaques. Science (2017) 358(6359):85–90. 10.1126/science.aan8630 28931639 PMC5978417

[148] Julg B Liu PT Wagh K Fischer WM Abbink P Mercado NB. Protection Against a Mixed SHIV Challenge by a Broadly Neutralizing Antibody Cocktail. Sci Transl Med (2017) 9(408):eaao4235. 10.1126/scitranslmed.aao4235 28931655 PMC5747528

[149] Mendoza P Gruell H Nogueira L Pai JA Butler AL Millard K. Combination Therapy With anti-HIV-1 Antibodies Maintains Viral Suppression. Nature (2018) 561(7724):479–84. 10.1038/s41586-018-0531-2 PMC6166473 30258136

[150] Borducchi EN Liu J Nkolola J Cadena AM Yu WH Fischinger S. (2018). Antibody and TLR7 Agonist Delay Viral Rebound in SHIV-Infected Monkeys, *N Engl J Med* 563(7731):360–4. 10.1038/s41586-018-0600-6 PMC6237629 30283138

[151] Lopez-Huertas MR Gutierrez C Madrid-Elena N Hernandez-Novoa B Olalla-Sierra J Plana M. Prolonged Administration of Maraviroc Reactivates Latent HIV In Vivo But it Does Not Prevent Antiretroviral-Free Viral Rebound. Sci Rep (2020) 10(1):22286. 10.1038/s41598-020-79002-w 33339855 PMC7749169

[152] Sarabia I Huang SH Ward AR Jones RB Bosque A. The Intact non-Inducible Latent Hiv-1 Reservoir is Established in an In Vitro Primary Tcm Cell Model of Latency. J Virol (2021). 10.1128/JVI.01297-20 PMC8092701 33441346

[153] Kaiser J. Gene Therapy Trials for Sickle Cell Disease Halted After Two Patients Develop Cancer (2021) 95(7):e01297–20. Available at: https://www.sciencemag.org/news/2021/02/gene-therapy-trials-sickle-cell-disease-halted-after-two-patients-develop-cancer.

[154] Maldini CR Gayout K Leibman RS Dopkin DL Mills JP Shan X. Hiv-Resistant and HIV-Specific Car-Modified Cd4(+) T Cells Mitigate Hiv Disease Progression and Confer Cd4(+) T Cell Help In Vivo. Mol Ther (2020) 28(7):1585–99. 10.1016/j.ymthe.2020.05.012 PMC7335752 32454027

